# Supplementary material for: Invasive pneumococcal disease in Latin America and the Caribbean: Serotype distribution, disease burden, and impact of vaccination. A systematic review and meta-analysis
Source: PLoS One. 2024 Jun 27;19(6):e0304978. doi: 10.1371/journal.pone.0304978 (PMC11210815; doi:10.1371/journal.pone.0304978)
Supplement: S1 File — (DOCX) [file pone.0304978.s002.docx]

# **Supporting information 2**

**S2 Search strategy**

**S2 Search strategy**

1. Name of the Database: Pubmed (MEDLINE)

Date of search: 27/12/2022

| Search | Query |
| --- | --- |
| #16 | #13 AND #14 Filters: from 2000/1/1 - 3000/12/12 |
| #15 | #13 AND #14 |
| #14 | (Americas[Majr] OR Latin America[Mesh] OR Latin America*[tiab] OR Latinamerica*[tiab] OR Latinoamerica*[tiab] OR Hispanoamerica*[tiab] OR Iberoamerica*[tiab] OR Ibero Americ*[tiab] OR Panamerican*[tiab] OR Central America[Mesh] OR Central America*[tiab] OR Centroamerica*[tiab] OR Mesoamerica*[tiab] OR Meso America*[tiab] OR Middle America*[tiab] OR South America[Mesh] OR South America*[tiab] OR Southamerica*[tiab] OR Sudamerica*[tiab] OR "America del Sur"[tiab] OR Caribbean Region[Mesh] OR Caribbean[tiab] OR Caribe*[tiab] OR West Indies[Mesh] OR West Indi*[tiab] OR Antill*[tiab] OR Indians, South American[Mesh] OR Indians, Central American[Mesh] OR Amerindian*[tiab] OR Indians[tiab] OR American Indian*[tiab] OR Native America*[tiab] OR Patagoni*[tiab] OR Andes[tiab] OR Andean*[tiab] OR Amazon*[tiab] OR Anguilla[ad] OR Anguill*[tiab] OR Anguilla[pl] OR "Antigua and Barbuda"[ad] OR "Antigua and Barbuda"[tiab] OR "Antigua and Barbuda"[pl] OR Argentin*[ad] OR Argentin*[tiab] OR Argentina[pl] OR Bahama*[ad] OR Baham*[tiab] OR Bahama*[pl] OR Bermud*[ad] OR Bermud*[tiab] OR Bermud*[pl] OR Bolivia*[ad] OR Bolivia*[tiab] OR Bolivia[pl] OR Brazil*[ad] OR Brasil*[ad] OR Brazil*[tiab] OR Brasil*[tiab] OR Brazil[pl] OR Cayman*[ad] OR Cayman*[tiab] OR Cayman*[pl] OR Curaçao[ad] OR Curaçao[tiab] OR Curaçao[pl] OR Colombia*[ad] OR Colombia*[tiab] OR Colombia[pl] OR Chile*[ad] OR Chile*[tiab] OR Chile[pl] OR Ecuador*[ad] OR Ecuator*[ad] OR Ecuador*[tiab] OR Ecuador[pl] OR Grenad*[ad] OR Grenad*[tiab] OR Grenad*[pl] OR Guadeloup*[ad] OR Guadeloup*[tiab] OR Guadeloup*[pl] OR Guiana*[ad] OR Guiana*[tiab] OR French Guiana[pl] OR Guyan*[ad] OR Guyan*[tiab] OR Guyana[pl] OR Paraguay*[ad] OR Paraguay*[tiab] OR Paraguay[pl] OR Peru*[ad] OR Peru*[tiab] OR Peru[pl] OR Surinam*[ad] OR Surinam*[tiab] OR Surinam*[pl] OR Uruguay*[ad] OR Uruguay*[tiab] OR Uruguay[pl] OR Venez*[ad] OR Venez*[tiab] OR Venezuela[pl] OR Belize*[ad] OR Belize*[tiab] OR Belize[pl] OR Costa Ric*[ad] OR Costarric*[ad] OR Costaric*[ad] OR Costa Ric*[tiab] OR Costarric*[tiab] OR Costaric*[tiab] OR Costa Rica[pl] OR Salvador*[ad] OR Salvador*[tiab] OR El Salvador[pl] OR Guatemal*[ad] OR Guatemal*[tiab] OR Guatemala[pl] OR Hondur*[ad] OR Hondur*[tiab] OR Honduras[pl] OR Martinique[ad] OR Martiniqu*[tiab] OR Martinique[pl] OR Nicaragu*[ad] OR Nicaragu*[tiab] OR Nicaragua[pl] OR Panam*[ad] OR Panam*[tiab] OR Panama[pl] OR Mexico[Mesh] OR Mexic*[ad] OR Mexic*[tiab] OR Mejic*[tiab] OR Mexico[pl] OR Montserrat[ad] OR Montserrat*[tiab] OR Montserrat[pl] OR Baham*[ad] OR Baham*[tiab] OR Bahamas[pl] OR Cuba*[ad] OR Cuba*[tiab] OR Cuba[pl] OR Dominic*[ad] OR Dominic*[tiab] OR Dominican Republic[pl] OR Haiti*[ad] OR Haiti*[tiab] OR Haiti[pl] OR Jamaic*[ad] OR Jamaic*[tiab] OR Jamaica[pl] OR Puerto Rico[Mesh] OR Puerto Ric*[tiab] OR Puertorric*[tiab] OR Puertoric*[tiab] OR Saint Kitts[ad] OR Saint Kitts[tiab] OR Saint Kitts[pl] OR "Trinidad and Tobago"[tiab] OR "Trinidad and Tobago"[ad] OR "Trinidad and Tobago"[pl]) |
| #13 | #1 OR #2 OR #3 OR #4 OR #5 OR #6 OR #7 OR #8 OR #9 OR #10 OR #11 OR #12 |
| #12 | PPV23[tiab] |
| #11 | PCV 15[tiab] |
| #10 | PCV 13[tiab] |
| #9 | PCV 10[tiab] |
| #8 | Pneumovax[tiab] |
| #7 | PnuImune Vaccine*[tiab] |
| #6 | Pnu-Imune Vaccine*[tiab] |
| #5 | Pneumococcal Vaccines[Mesh] |
| #4 | IPD[tiab] |
| #3 | Pneumococc*[tiab] |
| #2 | Streptococcus Pneumoniae[Mesh] |
| #1 | Pneumococcal Infections[Mesh] |

1. Name of Database: EMBase (OVID)

Date of search: 27/12/2022

Embase <1974 to 2022 December 23>

| # | Query |
| --- | --- |
| 1 | exp pneumococcal infection/ |
| 2 | exp Streptococcus pneumoniae/ |
| 3 | Pneumococc*.ti,ab. |
| 4 | IPD.ti,ab. |
| 5 | exp Pneumococcus vaccine/ |
| 6 | (Pnu-Imune adj3 Vaccine*).ti,ab. |
| 7 | (PnuImune adj3 Vaccine*).ti,ab. |
| 8 | Pneumovax.ti,ab. |
| 9 | PCV-10.ti,ab. |
| 10 | PCV-13.ti,ab. |
| 11 | PCV-15.ti,ab. |
| 12 | PPV23.ti,ab. |
| 13 | or/1-12 83268 |
| 14 | exp Latin America/ or exp South America/ or exp Central America/ or (Latin adj1 America*).ti,ab. or Latinamerica*.ti,ab. or Latinoamerica*.ti,ab. or Hispanoamerica.ti,ab. or Iberoamerica*.ti,ab. or (Ibero adj1 Americ*).ti,ab. or Panamerica*.ti,ab. or (South adj1 America*).ti,ab. or Southamerica*.ti,ab. or Sudamerica*.ti,ab. or (America adj1 Sur).ti,ab. or (Central adj1 America*).ti,ab. or Centroamerica*.ti,ab. or Mesoamerica*.ti,ab. or (Meso adj1 America*).ti,ab. or (Middle adj1 America*).ti,ab. or exp Caribbean Islands/ or Caribbean*.ti,ab. or Caribe*.ti,ab. or (West adj1 Indi*).ti,ab. or Antill*.ti,ab. or exp American indian/ or Amerindian*.ti,ab. or Indians.ti,ab. or (Native adj1 America*).ti,ab. or Patagoni*.ti,ab. or Andes.ti,ab. or Andean*.ti,ab. or Amazon*.ti,ab. or exp Argentina/ or Argentin*.ti,ab. or exp Bolivia/ or Bolivia*.ti,ab. or exp Brazil/ or Brazil*.ti,ab. or Brasil*.ti,ab. or exp Colombia/ or Colombia*.ti,ab. or exp Chile/ or Chile*.ti,ab. or exp Ecuador/ or Ecuador*.ti,ab. or exp French Guiana/ or Guiana*.ti,ab. or exp Guyana/ or Guyan*.ti,ab. or exp Paraguay/ or Paraguay*.ti,ab. or exp Peru/ or Peru*.ti,ab. or exp Suriname/ or Surinam*.ti,ab. or exp Uruguay/ or Uruguay*.ti,ab. or exp Venezuela/ or Venez*.ti,ab. or exp Belize/ or Beliz*.ti,ab. or exp Costa Rica/ or (Costa adj1 Rica).ti,ab. or Costarric*.ti,ab. or Costaric*.ti,ab. or exp El salvador/ or Salvador*.ti,ab. or exp Guatemala/ or Guatemal*.ti,ab. or exp Honduras/ or Hondur*.ti,ab. or exp Nicaragua/ or Nicaragu*.ti,ab. or exp Panama/ or Panam*.ti,ab. or exp Mexico/ or Mexic*.ti,ab. or exp Cuba/ or Cuba*.ti,ab. or exp Dominican Republic/ or Dominica*.ti,ab. or exp Haiti/ or Haiti*.ti,ab. or exp Jamaica/ or Jamaic*.ti,ab. or exp Puerto Rico/ or (Puerto adj1 Ric*).ti,ab. or Puertoric*.ti,ab. or Puertorric*.ti,ab. |
| 15 | 13 and 14 |
| 16 | limit 15 to yr="2000 -Current" |

1. Name of the Database: EconLIT (OVID)

Date of search: 27/12/2022

Econlit <1886 to December 15, 2022>

| # | Query |
| --- | --- |
| 1 | Pneumococc*.mp. |
| 2 | IPD.ti,ab. |
| 3 | (Pneumococ* adj3 Vaccin*).mp. |
| 4 | (Pnu-Imune adj3 Vaccine*).mp. |
| 5 | (PnuImune adj3 Vaccine*).mp. |
| 6 | Pneumovax.mp. |
| 7 | PCV-10.ti,ab. |
| 8 | PCV-13.ti,ab. |
| 9 | PCV-15.ti,ab. |
| 10 | PPV23.ti,ab. |
| 11 | or/1-10 97 |
| 12 | ((Latin adj1 America*) or Latinamerica* or Latinoamerica* or Latin* or Hispanic Americans or Iberoamerica* or (Ibero adj1 Americ*) or Panamerican* or (Central adj1 America*) or Centroamerica* or Mesoamerica* or (Meso adj1 America*) or (Middle adj1 America*) or (South adj1 America*) or Southamerica* or Sudamerica* or (America adj3 Sur) or Caribbean or Caribe* or (West adj1 Indi*) or Antill* or Amerindian* or Indians or (American adj1 Indian*) or (Native* adj1 America*) or Patagoni* or Andes or Andean* or Amazon* or Argentin* or Bolivia* or Brazil* or Brasil* Colombia* or Colombia* or Colombia or Chile* or Ecuador* or Guiana* or Guyan* or Guyan* or Paraguay* or Paraguay* or Peru* or Surinam* or Surinam* or Uruguay* or Venez* or Belize* or Costa Ric* or Costarric* or Costaric* or (Costa adj1 Ric*) or Costarric* or Salvador* or Salvador* or Salvador or Guatemal* or Guatemal* or Guatemal a or Hondur* or Nicaragu* Panam* or Mexic* or Cuba* or Dominic* or Dominic* or Haiti* or Jamaic* or Puerto Ric* or Puertorric* or Puertoric*).ti,ab. |
| 13 | 11 and 12 |

1. Name of the Database: CINAHL (EBSCO)

Date of search: 27/12/2022

| # | Query |
| --- | --- |
| S18 | S14 AND S17 Limiters - Published Date: 20000101-20221231 |
| S17 | S15 OR S16 |
| S16 | AB (Latin N1 America*) OR Latinamerica* OR Latinoamerica* OR Latin* OR Hispanic Americans OR Iberoamerica* OR (Ibero N1 Americ*) OR Panamerican* OR (Central N1 America*) OR Centroamerica* OR Mesoamerica* OR (Meso N1 America*) OR (Middle N1 America*) OR (South N1 America*) OR Southamerica* OR Sudamerica* OR (America N1 Sur) OR Caribbean OR Caribe* OR (West N1 Indi*) OR Antill* OR Amerindian* OR Indians OR (American N1 Indian*) OR (Native N1 America*) OR Patagoni* OR Andes OR Andean* OR Amazon* OR Argentin* OR Bolivia* OR Brazil* OR Brasil* Colombia* OR Colombia* OR Colombia OR Chile* OR Ecuador* OR Guiana* OR Guyan* OR Guyan* OR Paraguay* OR Paraguay* OR Peru* OR Surinam* OR Surinam* OR Uruguay* OR Venez* OR Belize* OR (Costa N1 Ric*) OR Costarric* OR Costaric* OR Costa Ric* OR Costarric* OR Salvador* OR Salvador* OR Guatemal* OR Guatemal* OR Guatemala OR Hondur* OR Nicaragu* OR Panam* OR Mexic* OR Cuba* OR Dominic* OR Dominic* OR Haiti* OR Jamaic* OR (Puerto N1 Ric*) OR Puertorric* OR Puertoric* |
| S15 | TI (Latin N1 America*) OR Latinamerica* OR Latinoamerica* OR Latin* OR Hispanic Americans OR Iberoamerica* OR (Ibero N1 Americ*) OR Panamerican* OR (Central N1 America*) OR Centroamerica* OR Mesoamerica* OR (Meso N1 America*) OR (Middle N1 America*) OR (South N1 America*) OR Southamerica* OR Sudamerica* OR (America N1 Sur) OR Caribbean OR Caribe* OR (West N1 Indi*) OR Antill* OR Amerindian* OR Indians OR (American N1 Indian*) OR (Native N1 America*) OR Patagoni* OR Andes OR Andean* OR Amazon* OR Argentin* OR Bolivia* OR Brazil* OR Brasil* Colombia* OR Colombia* OR Colombia OR Chile* OR Ecuador* OR Guiana* OR Guyan* OR Guyan* OR Paraguay* OR Paraguay* OR Peru* OR Surinam* OR Surinam* OR Uruguay* OR Venez* OR Belize* OR (Costa N1 Ric*) OR Costarric* OR Costaric* OR Costa Ric* OR Costarric* OR Salvador* OR Salvador* OR Guatemal* OR Guatemal* OR Guatemala OR Hondur* OR Nicaragu* OR Panam* OR Mexic* OR Cuba* OR Dominic* OR Dominic* OR Haiti* OR Jamaic* OR (Puerto N1 Ric*) OR Puertorric* OR Puertoric* |
| S14 | S1 OR S2 OR S3 OR S4 OR S5 OR S6 OR S7 OR S8 OR S9 OR S10 OR S11 OR S12 OR S13 |
| S13 | TI PPV23 OR AB PPV23 |
| S12 | TI PCV-15 OR AB PCV-15 |
| S11 | TI PCV-13 OR AB PCV-13 |
| S10 | TI PCV-10 OR AB PCV-10 |
| S9 | TI Pneumovax OR AB Pneumovax |
| S8 | TI (PnuImune N1 Vaccin*) OR AB (PnuImune N1 Vaccin*) |
| S7 | TI (Pnu-Imune N1 Vaccin*) OR AB (Pnu-Imune N1 Vaccin*) |
| S6 | TI (Pneumococcal N1 Vaccin*) OR AB (Pneumococcal N1 Vaccin*) |
| S5 | (MH "Pneumococcal Vaccine") |
| S4 | TI IPD OR AB IPD |
| S3 | TI Pneumococc* OR AB Pneumococc* |
| S2 | (MH "Pneumonia, Bacterial+") |
| S1 | (MH "Pneumococcal Infections+") |

1. Name of the Database: Global Health (OVID)

Date of search: 27/12/2022

Global Health <1910 to 2022 Week 51>

| # | Query |
| --- | --- |
| 1 | exp Streptococcus pneumoniae/ |
| 2 | Pneumococc*.ti,ab. |
| 3 | IPD.ti,ab. |
| 4 | (Pnu-Imune adj3 Vaccine*).ti,ab. |
| 5 | (PnuImune adj3 Vaccine*).ti,ab. |
| 6 | Pneumovax.ti,ab. |
| 7 | PCV-10.ti,ab. |
| 8 | PCV-13.ti,ab. |
| 9 | PCV-15.ti,ab. |
| 10 | PPV23.ti,ab. |
| 11 | or/1-10 23259 |
| 12 | exp Latin America/ or exp South America/ or exp Central America/ or (Latin adj1 America*).ti,ab. or Latinamerica*.ti,ab. or Latinoamerica*.ti,ab. or Hispanoamerica.ti,ab. or Iberoamerica*.ti,ab. or (Ibero adj1 Americ*).ti,ab. or Panamerica*.ti,ab. or (South adj1 America*).ti,ab. or Southamerica*.ti,ab. or Sudamerica*.ti,ab. or (America adj1 Sur).ti,ab. or (Central adj1 America*).ti,ab. or Centroamerica*.ti,ab. or Mesoamerica*.ti,ab. or (Meso adj1 America*).ti,ab. or (Middle adj1 America*).ti,ab. or exp Caribbean Islands/ or Caribbean*.ti,ab. or Caribe*.ti,ab. or (West adj1 Indi*).ti,ab. or Antill*.ti,ab. or exp American indian/ or Amerindian*.ti,ab. or Indians.ti,ab. or (Native adj1 America*).ti,ab. or Patagoni*.ti,ab. or Andes.ti,ab. or Andean*.ti,ab. or Amazon*.ti,ab. or exp Argentina/ or Argentin*.ti,ab. or exp Bolivia/ or Bolivia*.ti,ab. or exp Brazil/ or Brazil*.ti,ab. or Brasil*.ti,ab. or exp Colombia/ or Colombia*.ti,ab. or exp Chile/ or Chile*.ti,ab. or exp Ecuador/ or Ecuador*.ti,ab. or exp French Guiana/ or Guiana*.ti,ab. or exp Guyana/ or Guyan*.ti,ab. or exp Paraguay/ or Paraguay*.ti,ab. or exp Peru/ or Peru*.ti,ab. or exp Suriname/ or Surinam*.ti,ab. or exp Uruguay/ or Uruguay*.ti,ab. or exp Venezuela/ or Venez*.ti,ab. or exp Belize/ or Beliz*.ti,ab. or exp Costa Rica/ or (Costa adj1 Rica).ti,ab. or Costarric*.ti,ab. or Costaric*.ti,ab. or exp El salvador/ or Salvador*.ti,ab. or exp Guatemala/ or Guatemal*.ti,ab. or exp Honduras/ or Hondur*.ti,ab. or exp Nicaragua/ or Nicaragu*.ti,ab. or exp Panama/ or Panam*.ti,ab. or exp Mexico/ or Mexic*.ti,ab. or exp Cuba/ or Cuba*.ti,ab. or exp Dominican Republic/ or Dominica*.ti,ab. or exp Haiti/ or Haiti*.ti,ab. or exp Jamaica/ or Jamaic*.ti,ab. or exp Puerto Rico/ or (Puerto adj1 Ric*).ti,ab. or Puertoric*.ti,ab. or Puertorric*.ti,ab. |
| 13 | 11 and 12 |

1. Name of the Database: LILACS (BVS Eng)

Date of search: 27/12/2022

| Database: | LILACS |
| --- | --- |
| Search on: | (MH Pneumococcal Infections OR MH Streptococcus Pneumoniae OR Pneumococ$ OR Neumococ$ OR IPD OR ENI OR MH Pneumococcal Vaccines O Pnu-Imune OR PnuImune OR Pneumovax OR PCV-10 OR PCV-13 OR PCV-15 OR PPV23) [Words] and 2000 OR 2001 OR 2002 OR 2003 OR 2004 OR 2005 OR 2006 OR 2007 OR 2008 OR 2009 OR 2010 OR 2011 OR 2012 OR 2013 OR 2014 OR 2015 OR 2016 OR 2017 OR 2018 OR 2019 OR 2020 OR 2021 OR 2022 [Country, year publication] |

1. Name of the Database: Web of Science.

Date of search: 27/12/2022

| Web of Science Core Collection for: |
| --- |
| (TS=Pneumococcal Infections OR TS=Streptococcus Pneumoniae OR TI=Pneumococc* OR AB= Pneumococc* OR TI=IPD OR AB=IPD OR TS=Pneumococcal Vaccines OR TI=Pnu-Imune OR AB= Pnu-Imune OR TI=PnuImune OR AB=PnuImune OR TI=Pneumovax OR AB=Pneumovax OR TI=PCV-10 OR AB=PCV-10 OR TI=PCV-13 OR AB=PCV-13 OR TI=PCV 15 OR AB=PCV 15 OR TI=PPV23 OR AB=PPV23) AND (TS=Latin America OR TI=(Latin NEAR/1 America*) OR AB=(Latin NEAR/1 America*) OR ALL=Latinamerica* OR ALL=Latinoamerica* OR ALL=Hispanoamerica* OR ALL=Iberoamerica* OR TI=(Ibero NEAR/1 America*) OR AB=(Ibero NEAR/1 America*) OR ALL=Panamerican* OR TS=Central America OR TI=(Central NEAR/1 America*) OR AB=(Central NEAR/1 America*) OR ALL=Centroamerica* OR ALL=Mesoamerica* OR TI=(Meso NEAR/1 America*) OR AB=(Meso NEAR/1 America*) OR TI=(Middle NEAR/1 America*) OR AB=(Middle NEAR/1 America*) OR TS=South America OR TI=(South NEAR/1 America*) OR AB=(South NEAR/1 America*) OR ALL=Southamerica* OR ALL=Sudamerica* OR TI=(America NEAR/1 Sur) OR AB=(America NEAR/1 Sur) OR TS=Caribbean Region OR ALL=Caribbean OR ALL=Caribe* OR TS=West Indies OR TI=(West NEAR/1 Indi*) OR AB=(West NEAR/1 Indi*) OR ALL=Antill* OR TS=Indians, South American OR TS=Indians, Central American OR ALL=Amerindian* OR TI=(America* NEAR/3 Indian*) OR AB=(America* NEAR/3 Indian*) OR TI=(Native NEAR/1 America*) OR AB=(Native NEAR/1 America*) OR ALL=Patagoni* OR ALL=Andes OR ALL=Andean* OR ALL=Amazon* OR ALL=Anguill* OR TI=(Antigua NEAR/1 Barbuda) OR AB=(Antigua NEAR/1 Barbuda) OR ALL=Argentin* OR ALL=Baham* OR ALL=Bermud* OR ALL=Bolivia* OR ALL=Brazil* OR ALL=Brasil* OR ALL=Cayman* OR ALL=Curaçao OR ALL=Colombia* OR ALL=Chile* OR ALL= Ecuador* OR ALL=Grenad* OR ALL=Guadeloup* OR ALL=Guiana* OR ALL=Guyan* OR ALL=Paraguay* OR ALL=Peru* OR ALL=Surinam* OR ALL=Uruguay* OR ALL=Venez* OR ALL=Belize* OR TI=(Costa NEAR/1 Ric*) OR AB=(Costa NEAR/1 Ric*) OR ALL=Costarric* OR ALL=Costaric* OR ALL=Salvador* OR ALL=Guatemal* OR ALL=Hondur* OR ALL=Martiniqu* OR ALL=Nicaragu* OR ALL=Panam* OR TS=Mexico OR ALL=Mexic* OR ALL=Montserrat* OR ALL=Cuba* OR ALL=Dominic* OR ALL=Haiti* OR ALL=Jamaic* OR TS=Puerto Rico OR TI=(Puerto NEAR/1 Ric*) OR AB=(Puerto NEAR/1 Ric*) OR ALL=Puertorric* OR TI=(Saint NEAR/1 Kitts) OR AB=(Saint NEAR/1 Kitts) OR TI=(Trinidad NEAR/1 Tobago) OR AB=(Trinidad NEAR/1 Tobago)) and 2000 or 2001 or 2002 or 2003 or 2004 or 2005 or 2023 or 2022 or 2021 or 2020 or 2019 or 2018 or 2017 or 2016 or 2015 or 2014 or 2013 or 2012 (Publication Years) |

**S1 Table 2. List of excluded studies at full text screening stage**

| Author, year | Reason for exclusion |
| --- | --- |
| Agudelo 2005 | Duplicate |
| Agudelo 2021 | Duplicate |
| Alarcon 2021 | Duplicate |
| Almeida 2021 | Duplicate |
| Alves Cardozo 2014 | Duplicate |
| Andrade 2012 | Duplicate |
| Andrade 2016 | Not enough information |
| Arguedas 2012 | Duplicate |
| Asturias 2003 | Duplicate |
| Bautista-Mátquez 2013 | Wrong patient population |
| Benavides 2012 | Duplicate |
| Brandileone 2018 | Duplicate |
| Carnalla-Barajas 2017 | Wrong patient population |
| Catañeda 2009 | Duplicate |
| Cazentini Medeiros 2016 | Duplicate |
| Chacon Cruz 2012 | Duplicate |
| Chacon Cruz 2014 | Duplicate |
| Chacon Cruz 2016 | Duplicate |
| Chiou 2008 | Wrong outcomes |
| Costa Rica 2013 | Duplicate |
| Davalos 2016 | Duplicate |
| Di Fabio 2001 | Duplicate |
| Dickinson Meneses 2002 | Duplicate |
| dos Santos 2011 | Duplicate |
| Duarte 2022 | Wrong patient population |
| Echániz-Avilés 2014 | Wrong patient population |
| Echániz-Avilés 2019 | Duplicate |
| Feris Iglesias 2014 | Duplicate |
| Ferrer 2018 | Wrong setting |
| Firacative 2009 | Duplicate |
| Gabastou 2008 | Duplicate |
| Gagetti 2018 | Duplicate |
| Gaiano 2013 | Duplicate |
| García Quesada 2021 | Duplicate |
| Gómez Rodrígues 2006 | Wrong outcomes |
| Gómez-Barreto 2000 | Duplicate |
| Grando 2015 | Wrong setting |
| Grenón 2014 | Duplicate |
| Guevara-Duncan 2008 | Wrong patient population |
| Hidalgo 2011 | Duplicate |
| Hortal 2012 | Duplicate |
| Inostroza 2001 | Duplicate |
| Izquierdo 2005 | Wrong outcomes |
| Jimbo Sotomayor 2020 2021 | Wrong patient population |
| Ko 2000 | Duplicate |
| Lagos 2002 | Duplicate |
| Leal 2017 | Duplicate |
| Leal Castro 2019 | Duplicate |
| Lovera 2005 | Duplicate |
| Mantese 2003 | Duplicate |
| Moreno-Camacho 2021 | Duplicate |
| Morera Álvarez 2019 | Duplicate |
| Mott 2019 | Wrong patient population |
| Palacios 2017 | Duplicate |
| Pérez 2013 | Duplicate |
| Pérez Rodríguez 2011 | Duplicate |
| Pinheiro 2012 | Wrong patient population |
| Pinto 2016 | Duplicate |
| Pírez García 2011 | Duplicate |
| Ryoka Miyao Yoshioka 2012 | Duplicate |
| Salmeron Olsina 2018 | Duplicate |
| Sartori 2013 | Duplicate |
| Soto Nogueron 2016 | Wrong patient population |
| Soto-Noguerón 2018 | Wrong patient population |
| Tomczyk 2018 | Duplicate |
| Torres Cardoso 2017 | Wrong patient population |
| Verani 2015 | Wrong outcomes |
| Willis 2012 | Not enough information |

**S1 Table 3.** **Characteristics of included studies (n= 155)**

| **Author and year of publication** | **Country** | **Study start date dd/mm/yyyy** | **Study ending date dd/mm/yyyy** | **Study design** | **Age range** | **Sample size** | **Outcomes^#^** |
| --- | --- | --- | --- | --- | --- | --- | --- |
| Abate 2014*[1] | Argentina | 01/01/1993 | 31/12/2011 | Cross sectional | <18y | 537 | IPD: mortality, serotypes |
| Altclas 2004*[2] | Argentina | 01/01/1993 | 30/06/1998 | Cross sectional | All ages | 107 | Bacteremia: mortality |
| Bakir 2003*[3] | Argentina | 01/01/1993 | 31/12/1999 | Cross sectional | <18y | 274 | IPD: mortality |
| Barboza 2002*[4] | Argentina | 01/01/1988 | 31/12/1998 | Cross sectional | ≥18y | 87 | Meningitis: prevalence, mortality |
| Benitez 2017*[5] | Argentina | 01/05/2013 | 30/04/2014 | Cross sectional | <14y | 23 | IPD: mortality |
| Berberian 2014*[6] | Argentina | 01/01/1999 | 31/12/2010 | Cross sectional | <18y | 111 | Meningitis: mortality |
| Corbacho-Re 2020*[7] | Argentina | NR | NR | Case series | ≥18y | 274 | Pneumonia: prevalence |
| Fonaroff 2014*[8] | Argentina | 01/01/2004 | 31/12/2010 | Case series | ≥15y | 93 | Pneumonia: mortality |
| Gagetti 2017*[9] | Argentina | 01/01/1993 | 31/12/2014 | Cross sectional/  Surveillance | <5y | 4391 | IPD: prevalence, serotypes |
| Gagetti 2021*[10] | Argentina | 01/01/1998 | 31/12/2013 | Cross sectional/  Surveillance | <5y | 1713 | IPD: serotypes |
| Gentile 2003*[11] | Argentina | 01/01/1995 | 30/12/2000 | Case series | ≥18y | 101 | Pneumonia: incidence, mortality |
| Gentile 2018a*[12] | Argentina | 01/01/2007 | 31/12/2014 | Cross sectional/  Surveillance | <18y | 297 | Pneumonia: mortality, serotypes |
| Gentile 2018b*[13] | Argentina | 01/01/2012 | 31/12/2017 | Cross sectional | <18y | 135 | Pneumonia: prevalence |
| Grenón 2005*[14] | Argentina | 01/06/1998 | 30/06/2001 | Cross sectional | <14y | 101 | IPD: serotypes |
| Grenón 2014*[15] | Argentina | 01/01/1994 | 31/12/2009 | Case series | <14y | 167 | Meningitis: incidence, serotypes |
| Lopez 2018*[16] | Argentina | 01/07/2009 | 31/12/2013 | Non-comparative cohort | <5y | 1528 | IPD: prevalence, serotypes |
| Mathurin 2008*[17] | Argentina | 01/07/2014 | 31/12/2007 | Prospective cohort | ≥18y | 64 | Bacteriemia: incidence, mortality |
| Mayoral 2008*[18] | Argentina | 01/01/2003 | 31/12/2005 | Case series | <5y | 76 | IPD: serotypes |
| Paganini 2001*[19] | Argentina | 01/01/1996 | 31/12/1998 | Cross sectional | <18y | 109 | Pneumonia: prevalence, mortality |
| Palma 2012*[20] | Argentina | 01/06/1997 | 30/05/2001 | Cross sectional | ≥18y | 118 | Pneumonia: prevalence, mortality |
| Paniagua 2007*[21] | Argentina | 01/01/2002 | 30/08/2006 | Cross sectional | <18y | 106 | Meningitis: prevalence |
| Pérez 2014*[22] | Argentina | 01/10/2008 | 30/09/2013 | Case series | <18t | 171 | Bacteriemia: mortality, serotypes |
| Reijtman 2011**[23] | Argentina | 01/05/2009 | 30/08/2010 | Case series | <18y | 89 | IPD: serotypes |
| Ruvinsky 2010*[24] | Argentina | 01/01/1994 | 31/12/2007 | Cross sectional/  Surveillance | <5y | 2205 | IPD: serotypes |
| Tregnaghi 2006*[25] | Argentina | 01/12/1999 | 30/11/2002 | Cross sectional/  Surveillance | <2y | 21903 | IPD: prevalence, incidence, mortality, serotypes |
| Zintgraff 2020*[26] | Argentina | 01/01/2013 | 31/12/2017 | Cross sectional/  Surveillance | ≥18y | 791 | IPD: serotypes |
| Alvares 2011*[27] | Brazil | 01/04/1999 | 30/04/2009 | Case series | All ages | 72 | Meningitis: mortality, serotypes |
| Azevedo 2016*[28] | Brazil | 01/01/2008 | 31/12/2012 | Cross sectional | All ages | 148 | Meningitis: incidence, mortality, serotypes |
| Barroso 2012*[29] | Brazil | 01/01/2000 | 31/12/2008 | Cross sectional/  Surveillance | All ages | 1272 | Meningitis: incidence, mortality, serotypes |
| Berezin 2002*[30] | Brazil | 01/01/1994 | 31/12/1999 | Case series | <18y | 55 | Meningitis: mortality, serotypes |
| Berezin 2007*[31] | Brazil | 01/06/1997 | 31/05/2001 | Case series | <5y | 625 | IPD: prevalence, serotypes |
| Berezin 2020*[32] | Brazil | 01/01/2005 | 31/12/2015 | Case series | <18y | 260 | IPD: mortality, serotypes |
| Blanco 2020*[33] | Brazil | 01/08/2008 | 31/12/2018 | Case series | <18y | 90 | Meningitis: prevalence, incidence, mortality |
| Brandileone 2003*[34] | Brazil | 01/01/1977 | 31/12/2000 | Cross sectional/  Surveillance | All ages | 4858 | IPD: serotypes |
| Brandileone 2021*[35] | Brazil | 01/01/2007 | 31/12/2019 | Cross sectional/  Surveillance | All ages | 11380 | IPD: serotypes |
| Caierao 2014*[36] | Brazil | 01/01/2007 | 31/12/2012 | Cross sectional | All ages | 325 | IPD: serotypes |
| Camargos 2020*[37] | Brazil | 01/01/1990 | 31/12/2017 | Cross sectional/  Surveillance | <5y | NR | Meningitis: mortality |
| Cassiolato 2019***[38] | Brazil | 01/01/2005 | 31/12/2017 | Cross sectional | All ages | 9854 | IPD: serotypes |
| Cazentini Medeiros  2017*[39] | Brazil | 01/01/1998 | 31/12/2013 | Cross sectional/  Surveillance | All ages | 796 | IPD: prevalence, serotypes |
| Christophe 2018*[40] | Brazil | 01/01/2013 | 01/06/2015 | Cross sectional | >50y | 102 | IPD: serotypes |
| da Silva 2010*[41] | Brazil | 01/01/2005 | 31/12/2008 | Case series | All ages | 168 | Meningitis: prevalence, mortality |
| dos Santos 2013*[42] | Brazil | 01/01/2006 | 30/09/2012 | Case series | All ages | 259 | IPD: incidence |
| Gomes de Oliveira  Magalhaes 2003*[43] | Brazil | 01/06/2000 | 30/05/2001 | Cross sectional/  Surveillance | <5y | 31 | IPD: serotypes |
| Gouveia 2011*[44] | Brazil | 01/12/1995 | 30/11/2005 | Case series | All ages | 548 | Meningitis: mortality, serotypes |
| Hirose 2015*[45] | Brazil | 01/01/1998 | 31/12/2011 | Non-comparative cohort | All ages | 1339 | Meningitis: incidence, mortality |
| Jarovsky 2017**[46] | Brazil | 01/01/2000 | 30/04/2017 | Case series | All ages | 561 | IPD: incidence, mortality |
| Laval 2006*[47] | Brazil | 01/05/2000 | 31/08/2001 | Cross sectional/  Surveillance | <5y | 773 | IPD: seotypes |
| Leite 2016*[48] | Brazil | 01/07/2010 | 31/12/2013 | Case series | All ages | 82 | IPD: serotypes |
| Levin 2003*[49] | Brazil | 01/07/1991 | 31/12/1994 | Cross sectional | All ages | 165 | IPD: prevalence, mortality |
| Mantese 2002*[50] | Brazil | 01/01/1987 | 31/01/2001 | Cross sectional | <18y | 415 | Meningitis: prevalence, mortality |
| Mantese 2009*[51] | Brazil | 01/04/1999 | 31/12/2008 | Case series | <5y | 142 | IPD: serotypes |
| Mendes Lages 2020*[52] | Brazil | 01/01/2005 | 31/12/2016 | Case series | <18y | 144 | IPD: incidence, serotypes |
| Menezes 2011*[53] | Brazil | 01/01/2000 | 31/12/2007 | Cross sectional/  Surveillance | All ages | 421 | Meningitis: incidence, serotypes |
| Mott 2014*[54] | Brazil | 01/01/2010 | 30/04/2012 | Cross sectional/  Surveillance | All ages | 159 | IPD: serotypes |
| Nascimento-Carvalho  2003*[55] | Brazil | 01/09/1997 | 31/05/2002 | Cross sectional/  Surveillance | <18y | 70 | IPD: serotypes |
| Neves Reis 2002*[56] | Brazil | 01/12/1995 | 30/11/1999 | Case series | All ages | 305 | Meningitis: incidence, mortality, serotypes, |
| Novaes 2011*[57] | Brazil | 01/01/2004 | 31/12/2006 | Cross sectional/  Surveillance | All ages | NR | IPD: mortality |
| Oliveira 2019*[58] | Brazil | 01/01/2005 | 31/12/2013 | Ecological study | All ages | 3963 | Meningitis: incidence |
| Pinto 2019*[59] | Brazil | 01/01/1990 | 31/12/2014 | Cross sectional | All ages | 783 | IPD: serotypes |
| Rocha Dullius 2018*[60] | Brazil | 01/01/2005 | 31/12/2016 | Cross sectional | NR | 118 | IPD: mortality, serotypes |
| Rossoni 2008*[61] | Brazil | 01/04/2001 | 30/08/2002 | Cross sectional | All ages | 436 | Meningitis: prevalence, mortality, serotypes |
| Soares dos Santo  2022*[62] | Brazil | 01/01/1996 | 31/12/2012 | Cross sectional/  Surveillance | NR | 917 | Meningitis: prevalence, mortality, serotypes |
| Veras 2007*[63] | Brazil | 01/11/2000 | 30/08/2004 | Case series | ≥18y | 79 | IPD: mortality, serotypes |
| Vieira 2007*[64] | Brazil | 01/01/1995 | 31/12/2004 | Case series | All ages | 232 | Meningitis: serotypes |
| Yoshioka 2011*[65] | Brazil | 01/01/2003 | 30/10/2008 | Cross sectional | <18y | 107 | Pneumonia: serotypes |
| Aguilera 2010*[66] | Chile | 01/01/2005 | 30/08/2006 | Case series | ≥18y | 56 | Bacteriemia: serotypes |
| Alvarado 2018*[67] | Chile | 01/01/2009 | 31/12/2015 | Ecological study | <5y | 169 | IPD: incidence, mortality, serotypes |
| Contreras 2002*[68] | Chile | 01/04/1994 | 30/05/1999 | Cross sectional | <18y | 78 | IPD: mortality, serotypes |
| Fica 2014*[69] | Chile | 01/01/2005 | 31/12/2010 | Case series | ≥18y | 59 | Pneumonia: mortality, serotypes |
| Inostroza 2007*[70] | Chile | 01/01/1994 | 31/11/2004 | Cross sectional/  Surveillance | All ages | 514 | IPD: incidence, serotypes |
| Abarca 2008*[71] | Chile | 01/05/2001 | 30/04/2002 | Cross sectional/  Surveillance | <2y | 4369 | IPD: prevalence, incidence, mortality, serotypes |
| Lagos 2008*[72] | Chile | 01/01/994 | 31/12/2007 | Cross sectional/  Surveillance | <18y | 2369 | IPD: incidence, mortality, serotypes |
| Maldonado 2007*[73] | Chile | 01/0!/2000 | 31/12/2006 | Cross sectional/  Surveillance | ≥18y | 1429 | IPD: serotypes |
| Rioseco 2004*[74] | Chile | 01/01/1997 | 31/08/2002 | Case series | ≥18y | 45 | Pneumonia: mortality |
| Rioseco 2018*[75] | Chile | 01/01/2010 | 31/12/2014 | Case series | ≥18y | 70 | Pneumonia: mortality, serotypes |
| Saldías 2011*[76] | Chile | 01/01/2002 | 31/12/2005 | Case series | ≥18y | 151 | Pneumonia: prevalence |
| Valenzuela 2014*[77] | Chile | 01/01/2007 | 31/12/2012 | Cross sectional/  Surveillance | All ages | 4829 | IPD: incidence, serotypes |
| Africano 2020*[78] | Colombia | 01/01/2012 | 31/12/2019 | Cross sectional | ≥18y | 310 | IPD: incidence, serotypes |
| Agudelo 2001*[79] | Colombia | 01/01/1994 | 31/12/2000 | Cross sectional/  Surveillance | <5y | 764 | IPD: serotypes |
| Agudelo 2002*[80] | Colombia | 01/01/1998 | 30/11/2001 | Cross sectional/  Surveillance | >5y | 343 | IPD: serotypes |
| Agudelo 2006*[81] | Colombia | 01/01/1994 | 31/12/2004 | Cross sectional/  Surveillance | All ages | 2022 | IPD: serotypes, antimicrobial susceptibility |
| Caceres 2018*[82] | Colombia | 01/01/2005 | 31/12/2015 | Ecological study | All ages | 1056 | Meningitis: prevalence |
| Calderon 2014*[83] | Colombia | 01/01/2010 | 30/06/2011 | Cross sectional | ≥18y | 60 | IPD: mortality |
| Camacho Moreno  2020*[84] | Colombia | 01/01/2008 | 31/12/2017 | Case series | <18y | 463 | IPD: mortality, serotypes |
| Farfán-Albarracín  2022*[85] | Colombia | 01/01/2008 | 31/12/2019 | Case series | <18y | 81 | Meningitis: incidence, mortality, serotypes |
| Gutierrez-Tobar 2022*[86] | Colombia | 01/01/2008 | 31/12/2019 | Cross sectional/  Surveillance | <18y | 566 | Pneumonia: prevalence, incidence, serotypes |
| Leal Castro 2022*[87] | Colombia | 01/01/2011 | 31/12/2017 | Case series | ≥18y | 169 | IPD: mortality, serotypes |
| Moreno 2004*[88] | Colombia | 01/01/2000 | 31/12/2003 | Cross sectional/  Surveillance | <5y | 190 | IPD: serotypes |
| Narváez 2021*[89] | Colombia | 01/01/2012 | 31/01/2019 | Case series | ≥18y | 310 | IPD: mortality, serotypes |
| Parra 2014*[90] | Colombia | 01/01/2005 | 31/12/2010 | Cross sectional/  Surveillance | All ages | 1775 | IPD: serotypes |
| Parra 2017*[91] | Colombia | 01/01/1994 | 31/12/2013 | Cross sectional/  Surveillance | All ages | 4991 | IPD: serotypes |
| Rojas 2016*[92] | Colombia | 01/01/2008 | 15/01/2014 | Case series | <18y | 239 | IPD: mortality |
| Severiche-Bueno  2021*[93] | Colombia | 01/01/2007 | 31/12/2017 | Cross sectional/  Surveillance | All ages | 1670 | IPD: incidence, serotypes |
| Vela 2001*[94] | Colombia | 01/02/1994 | 31/12/1999 | Cross sectional/  Surveillance | <5y | 167 | IPD: serotypes |
| Barboza 2018**[95] | Costa Rica | 01/01/2009 | 31/12/2015 | Cross sectional | <2y | 76 | Meningitis: prevalence, mortality |
| Ulloa-Gutierrez 2003*[96] | Costa Rica | 01/01/1995 | 31/12/2001 | Case series | <14y | 132 | IPD: incidence, mortality |
| Vargas-Gutierrez  2015**[97] | Costa Rica | 01/01/2006 | 31/01/2014 | Case series | <14y | 121 | Pneumonia: prevalence, serotypes |
| Batista Caluff 2017*[98] | Cuba | 01/01/2011 | 31/12/2015 | Cross sectional | <5y | 42 | IPD: mortality, serotypes |
| Dickinson Meneses  2017*[99] | Cuba | 01/01/1998 | 31/12/2015 | Prospective cohort | <5y | 483 | Meningitis: incidence, mortality |
| Fonseca Hernandez  2017*[100] | Cuba | 01/01/2014 | 31/03/2016 | Case series | <5y | 37 | IPD: mortality, serotypes |
| Morera Álvarez 2019*[101] | Cuba | 01/01/2009 | 31/12/2015 | Cross sectional | <5y | 94 | IPD: incidence, mortality, serotypes |
| Pérez 2009*[102] | Cuba | 01/01/1998 | 31/12/2007 | Case series | All ages | 4798 | Meningitis: prevalence, mortality |
| Rodríguez Cutting  2017*[103] | Cuba | 01/01/2000 | 31/12/2014 | Case series | <18y | 707 | Pneumonia: prevalence |
| Toraño Peraza 2014*[104] | Cuba | 01/01/2007 | 31/12/2012 | Case series | All ages | 237 | Meningitis: serotypes |
| Toraño Peraza 2017*[105] | Cuba | 01/01/2013 | 31/12/2015 | Case series | <18y | 141 | IPD: serotypes |
| Ahmed 2021*[106] | Dominican Republic | 01/07/2009 | 30/06/2016 | Cross sectional/  Surveillance | <14y | 342 | Pneumonia: prevalence, serotypes |
| Jonnalagadda 2017*[107] | Ecuador | 01/02/2008 | 20/04/2010 | Cross sectional | <5y | 403 | Pneumonia: prevalence |
| Juliao 2021*[108] | Ecuador | 02/01/2005 | 31/12/2017 | Cross sectional/  Surveillance | <5y | NR | IPD: incidence, mortality |
| Elenga 2015*[109] | French Guiana | 01/01/2000 | 31/12/2010 | Case series | <18y | 60 | Meningitis: mortality, serotypes |
| Gaensbauer 2016*[110] | Guatemala | 01/10/1996 | 31/12/2007 | Case series | <5y | 452 | IPD: mortality, serotypes |
| Melgar 2019**[111] | Guatemala | 01/03/2016 | 31/03/2019 | Case series | All ages | 1119 | IPD: prevalence, mortality, serotypes  Meningitis: mortality |
| Trotman 2009*[112] | Jamaica | 01/01/1995 | 31/12/1999 | Case series | <5y | 25 | Meningitis: mortality |
| Willis 2018*[113] | Jamaica | 01/01/2008 | 31/12/2009 | Prospective cohort | All ages | 350 | IPD: serotypes |
| Willis 2019*[114] | Jamaica | 01/01/2008 | 31/12/2009 | Case series | All ages | 94 | IPD: mortality |
| Alves Cardozo 2008*[115] | LAC | 01/07/1998 | 31/12/2002 | Cross sectional | <5y | 240 | Pneumonia: mortality, serotypes |
| Andrade 2012*[116]] | LAC | 01/01/2007 | 31/12/2009 | Cross sectional/  Surveillance | <5y | 31571 | IPD: prevalence, serotypes |
| Hortal 2000b*[117] | LAC | 01/01/1993 | 20/09/1999 | Cross sectional/  Surveillance | <5y | 3393 | Pneumonia: mortality, serotypes |
| Moreno 2020*[118] | LAC | 01/01/2000 | 31/12/2015 | Cross sectional/  Surveillance | All ages | 185 | IPD: serotypes |
| Peltola 2021*[119] | LAC | 01/01/1995 | 31/12/2003 | Cross sectional | <18y | 654 | Meningitis: prevalence, mortality |
| Zemlickova 2005*[120] | LAC | 01/01/2000 | 31/12/2002 | Cross sectional/  Surveillance | <5y | 185 | IPD: serotypes |
| Arredondo-García  2011*[121] | Mexico | 01/02/2002 | 31/12/2005 | Cross sectional | <18y | 150 | IPD: mortality, serotypes |
| Chacon Cruz 2017**[122] | Mexico | 01/10/2005 | 31/03/2017 | Case series | <18y | 57 | IPD: mortality, serotypes |
| Chacon Cruz 2019*[123] | Mexico | 01/10/2005 | 31/01/2018 | Case series | <18y | 64 | Pneumonia: serotypes |
| Echaniz-Aviles 2015*[124] | Mexico | 01/01/1993 | 31/12/2012 | Cross sectional/  Surveillance | <5y | 1346 | IPD: prevalence, serotypes |
| Echaniz-Aviles 2019*[125] | Mexico | 01/01/2000 | 30/09/2015 | Case series | ≥18y | 96 | IPD: mortality, serotypes |
| Franco-Paredes  2008*[126] | Mexico | 01/01/1993 | 31/12/2003 | Case series | <18y | 218 | Meningitis: prevalence |
| Gómez-Barreto 2010*[127] | Mexico | 01/01/1997 | 31/08/2014 | Case series | <14y | 156 | IPD: mortality, serotypes |
| Zarco Marquez 2016*[128] | Mexico | 01/01/2007 | 31/12/2015 | Case series | ≥18y | 69 | IPD: mortality, serotypes |
| De León 2011*[129] | Panama | 01/01/2010 | 30/06/2011 | Case series | <18y | 23 | IPD: mortality, serotypes |
| Aranda 2014*[130] | Paraguay | 01/01/1993 | 30/06/2006 | Cross sectional | <18y | 394 | Meningitis: mortality, serotypes |
| Leon 2020*[131] | Paraguay | 01/01/2010 | 31/12/2018 | Cross sectional/  Surveillance | All ages | 793 | IPD: serotypes |
| Lovera 2011*[132] | Paraguay | 01/01/200 | 30/04/2010 | Case series | <18y | 46 | Meningitis: mortality, serotypes |
| Sanabria 2009*[133] | Paraguay | 01/01/2002 | 30/08/2007 | Cross sectional | <18y | 78 | IPD: mortality, serotypes |
| Castro 2017*[134] | Peru | 01/06/2009 | 30/06/2011 | Case series | ≥18y | 43 | IPD: mortality, serotypes |
| Castillo-Tokumori  2018**[135] | Peru | 01/11/2016 | 28/0272018 | Case series | <5y | 45 | IPD: prevalence, serotypes |
| Hawkins 2017*[136] | Peru | 01/01/2006 | 31/12/2011 | Cross sectional/  Surveillance | NR | 212 | IPD: serotypes |
| Luna-Muschi 2019*[137] | Peru | 01/01/2006 | 31/12/2011 | Cross sectional/  Surveillance | <18y | 159 | IPD: mortality, incidence, serotypes |
| Morales de Santa  Gadea 2003*[138] | Peru | 01/10/2000 | 31/12/2001 | Cross sectional | <5y | 1283 | IPD: mortality, serotypes |
| Rivera-Matos 2005*[139] | Puerto Rico | 01/01/2001 | 31/12/2001 | Cross sectional/  Surveillance | All ages | 192 | IPD: mortality, serotypes |
| Nurse Lucas 2016*[140] | Trinidad and Tobago | 01/01/1997 | 31/12/2013 | Cross sectional/  Surveillance | All ages | 83 | IPD: serotypes |
| Assandri 2015*[141] | Uruguay | 01/01/2001 | 31/12/2013 | Case series | <1y | 25 | IPD: mortality, serotypes |
| Camou 2003*[142] | Uruguay | 01/01/1994 | 31/12/2001 | Cross sectional/  Surveillance | <5y | 506 | IPD: mortality, serotypes |
| Cardinal-Fernández  2013*[143] | Uruguay | 01/01/2008 | 31/10/2010 | Prospective cohort | >18y | 192 | IPD: mortality, serotypes |
| Ferrari Castilla 2007*[144] | Uruguay | 01/01/1998 | 31/12/2004 | Cross sectional/  Surveillance | <18y | 512 | Pneumonia: serotypes |
| Gabarrot 2014*[145] | Uruguay | 01/01/2003 | 31/12/2012 | Retrospective cohort | All ages | 1887 | IPD: incidence, serotypes |
| Hortal 2000a*[146] | Uruguay | 01/01/1987 | 31/12/1997 | Cross sectional/  Surveillance | >5y | 228 | IPD: mortality, serotypes |
| Hortal 2007*[147] | Uruguay | 01/01/2001 | 31/05/2004 | Cross sectional/  Surveillance | <5y | 2034 | Pneumonia: prevalence, serotypes |
| Hortal 2008*[148] | Uruguay | 01/06/2000 | 31/12/2004 | Case series | <14y | 410 | Pneumonia: mortality, serotypes |
| Hortal 2014*[149] | Uruguay | 01/01/2009 | 31/12/2012 | Cross sectional/  Surveillance | <14y | 3677 | Pneumonia: prevalence, serotypes |
| Machado 2014*[150] | Uruguay | 01/01/2010 | 31/12/2010 | Case series | <18y | 43 | Pneumonia: mortality, serotypes |
| Machado 2020*[151] | Uruguay | 01/01/2009 | 31/12/2018 | Case series | <14y | 197 | Pneumonia: prevalence, mortality, serotypes |
| Pírez 2001*[152] | Uruguay | 01/09/1997 | 31/08/1998 | Case series | <5y | 1082 | Pneumonia: prevalence, mortality |
| Pírez 2014*[153] | Uruguay | 01/01/2003 | 31/12/2012 | Non-comparative cohort | <18y | 630 | Pneumonia: incidence |
| Pírez 2017*[154] | Uruguay | 01/01/2005 | 31/12/2014 | Case series | <18y | 52 | Meningitis: prevalence, mortality, serotypes |
| Pírez García 2008*[155] | Uruguay | 01/01/1998 | 31/12/2005 | Case series | <2y | 192 | Pneumonia: mortality, serotypes |

NR: Not Reported

*Full text; **Abstract/Poster

#IPD: Invasive Pneumococcal disease
