# Supplementary material for: Invasive pneumococcal disease in Latin America and the Caribbean: Serotype distribution, disease burden, and impact of vaccination. A systematic review and meta-analysis
Source: PLoS One. 2024 Jun 27;19(6):e0304978. doi: 10.1371/journal.pone.0304978 (PMC11210815; doi:10.1371/journal.pone.0304978)
Supplement: S2 File — (DOCX) [file pone.0304978.s003.docx]

# **Supporting information 3**

**S3. Table 1. Characteristics of included studies (n= 155)**

**S3 Table 2. Meta-analysis density incidence of IPD by age**

**S3 Table 3. Meta-analysis density incidence of meningitis in children under 5 years of age**

**S3 Table 4. Risk of bias assessment for cohort and cross sectional studies**

**S3 Table 5. Risk of bias assessment for case series studies**

**S1 Table 2. List of excluded studies at full text screening stage**

| Author, year | Reason for exclusion |
| --- | --- |
| Agudelo 2005 | Duplicate |
| Agudelo 2021 | Duplicate |
| Alarcon 2021 | Duplicate |
| Almeida 2021 | Duplicate |
| Alves Cardozo 2014 | Duplicate |
| Andrade 2012 | Duplicate |
| Andrade 2016 | Not enough information |
| Arguedas 2012 | Duplicate |
| Asturias 2003 | Duplicate |
| Bautista-Mátquez 2013 | Wrong patient population |
| Benavides 2012 | Duplicate |
| Brandileone 2018 | Duplicate |
| Carnalla-Barajas 2017 | Wrong patient population |
| Catañeda 2009 | Duplicate |
| Cazentini Medeiros 2016 | Duplicate |
| Chacon Cruz 2012 | Duplicate |
| Chacon Cruz 2014 | Duplicate |
| Chacon Cruz 2016 | Duplicate |
| Chiou 2008 | Wrong outcomes |
| Costa Rica 2013 | Duplicate |
| Davalos 2016 | Duplicate |
| Di Fabio 2001 | Duplicate |
| Dickinson Meneses 2002 | Duplicate |
| dos Santos 2011 | Duplicate |
| Duarte 2022 | Wrong patient population |
| Echániz-Avilés 2014 | Wrong patient population |
| Echániz-Avilés 2019 | Duplicate |
| Feris Iglesias 2014 | Duplicate |
| Ferrer 2018 | Wrong setting |
| Firacative 2009 | Duplicate |
| Gabastou 2008 | Duplicate |
| Gagetti 2018 | Duplicate |
| Gaiano 2013 | Duplicate |
| García Quesada 2021 | Duplicate |
| Gómez Rodrígues 2006 | Wrong outcomes |
| Gómez-Barreto 2000 | Duplicate |
| Grando 2015 | Wrong setting |
| Grenón 2014 | Duplicate |
| Guevara-Duncan 2008 | Wrong patient population |
| Hidalgo 2011 | Duplicate |
| Hortal 2012 | Duplicate |
| Inostroza 2001 | Duplicate |
| Izquierdo 2005 | Wrong outcomes |
| Jimbo Sotomayor 2020 2021 | Wrong patient population |
| Ko 2000 | Duplicate |
| Lagos 2002 | Duplicate |
| Leal 2017 | Duplicate |
| Leal Castro 2019 | Duplicate |
| Lovera 2005 | Duplicate |
| Mantese 2003 | Duplicate |
| Moreno-Camacho 2021 | Duplicate |
| Morera Álvarez 2019 | Duplicate |
| Mott 2019 | Wrong patient population |
| Palacios 2017 | Duplicate |
| Pérez 2013 | Duplicate |
| Pérez Rodríguez 2011 | Duplicate |
| Pinheiro 2012 | Wrong patient population |
| Pinto 2016 | Duplicate |
| Pírez García 2011 | Duplicate |
| Ryoka Miyao Yoshioka 2012 | Duplicate |
| Salmeron Olsina 2018 | Duplicate |
| Sartori 2013 | Duplicate |
| Soto Nogueron 2016 | Wrong patient population |
| Soto-Noguerón 2018 | Wrong patient population |
| Tomczyk 2018 | Duplicate |
| Torres Cardoso 2017 | Wrong patient population |
| Verani 2015 | Wrong outcomes |
| Willis 2012 | Not enough information |

**S3 Table 2.** **Characteristics of included studies (n= 155)**

| **Author and year of publication** | **Country** | **Study start date dd/mm/yyyy** | **Study ending date dd/mm/yyyy** | **Study design** | **Age range** | **Sample size** | **Outcomes^#^** |
| --- | --- | --- | --- | --- | --- | --- | --- |
| Abate 2014*[1] | Argentina | 01/01/1993 | 31/12/2011 | Cross sectional | <18y | 537 | IPD: mortality, serotypes |
| Altclas 2004*[2] | Argentina | 01/01/1993 | 30/06/1998 | Cross sectional | All ages | 107 | Bacteremia: mortality |
| Bakir 2003*[3] | Argentina | 01/01/1993 | 31/12/1999 | Cross sectional | <18y | 274 | IPD: mortality |
| Barboza 2002*[4] | Argentina | 01/01/1988 | 31/12/1998 | Cross sectional | ≥18y | 87 | Meningitis: prevalence, mortality |
| Benitez 2017*[5] | Argentina | 01/05/2013 | 30/04/2014 | Cross sectional | <14y | 23 | IPD: mortality |
| Berberian 2014*[6] | Argentina | 01/01/1999 | 31/12/2010 | Cross sectional | <18y | 111 | Meningitis: mortality |
| Corbacho-Re 2020*[7] | Argentina | NR | NR | Case series | ≥18y | 274 | Pneumonia: prevalence |
| Fonaroff 2014*[8] | Argentina | 01/01/2004 | 31/12/2010 | Case series | ≥15y | 93 | Pneumonia: mortality |
| Gagetti 2017*[9] | Argentina | 01/01/1993 | 31/12/2014 | Cross sectional/  Surveillance | <5y | 4391 | IPD: prevalence, serotypes |
| Gagetti 2021*[10] | Argentina | 01/01/1998 | 31/12/2013 | Cross sectional/  Surveillance | <5y | 1713 | IPD: serotypes |
| Gentile 2003*[11] | Argentina | 01/01/1995 | 30/12/2000 | Case series | ≥18y | 101 | Pneumonia: incidence, mortality |
| Gentile 2018a*[12] | Argentina | 01/01/2007 | 31/12/2014 | Cross sectional/  Surveillance | <18y | 297 | Pneumonia: mortality, serotypes |
| Gentile 2018b*[13] | Argentina | 01/01/2012 | 31/12/2017 | Cross sectional | <18y | 135 | Pneumonia: prevalence |
| Grenón 2005*[14] | Argentina | 01/06/1998 | 30/06/2001 | Cross sectional | <14y | 101 | IPD: serotypes |
| Grenón 2014*[15] | Argentina | 01/01/1994 | 31/12/2009 | Case series | <14y | 167 | Meningitis: incidence, serotypes |
| Lopez 2018*[16] | Argentina | 01/07/2009 | 31/12/2013 | Non-comparative cohort | <5y | 1528 | IPD: prevalence, serotypes |
| Mathurin 2008*[17] | Argentina | 01/07/2014 | 31/12/2007 | Prospective cohort | ≥18y | 64 | Bacteriemia: incidence, mortality |
| Mayoral 2008*[18] | Argentina | 01/01/2003 | 31/12/2005 | Case series | <5y | 76 | IPD: serotypes |
| Paganini 2001*[19] | Argentina | 01/01/1996 | 31/12/1998 | Cross sectional | <18y | 109 | Pneumonia: prevalence, mortality |
| Palma 2012*[20] | Argentina | 01/06/1997 | 30/05/2001 | Cross sectional | ≥18y | 118 | Pneumonia: prevalence, mortality |
| Paniagua 2007*[21] | Argentina | 01/01/2002 | 30/08/2006 | Cross sectional | <18y | 106 | Meningitis: prevalence |
| Pérez 2014*[22] | Argentina | 01/10/2008 | 30/09/2013 | Case series | <18t | 171 | Bacteriemia: mortality, serotypes |
| Reijtman 2011**[23] | Argentina | 01/05/2009 | 30/08/2010 | Case series | <18y | 89 | IPD: serotypes |
| Ruvinsky 2010*[24] | Argentina | 01/01/1994 | 31/12/2007 | Cross sectional/  Surveillance | <5y | 2205 | IPD: serotypes |
| Tregnaghi 2006*[25] | Argentina | 01/12/1999 | 30/11/2002 | Cross sectional/  Surveillance | <2y | 21903 | IPD: prevalence, incidence, mortality, serotypes |
| Zintgraff 2020*[26] | Argentina | 01/01/2013 | 31/12/2017 | Cross sectional/  Surveillance | ≥18y | 791 | IPD: serotypes |
| Alvares 2011*[27] | Brazil | 01/04/1999 | 30/04/2009 | Case series | All ages | 72 | Meningitis: mortality, serotypes |
| Azevedo 2016*[28] | Brazil | 01/01/2008 | 31/12/2012 | Cross sectional | All ages | 148 | Meningitis: incidence, mortality, serotypes |
| Barroso 2012*[29] | Brazil | 01/01/2000 | 31/12/2008 | Cross sectional/  Surveillance | All ages | 1272 | Meningitis: incidence, mortality, serotypes |
| Berezin 2002*[30] | Brazil | 01/01/1994 | 31/12/1999 | Case series | <18y | 55 | Meningitis: mortality, serotypes |
| Berezin 2007*[31] | Brazil | 01/06/1997 | 31/05/2001 | Case series | <5y | 625 | IPD: prevalence, serotypes |
| Berezin 2020*[32] | Brazil | 01/01/2005 | 31/12/2015 | Case series | <18y | 260 | IPD: mortality, serotypes |
| Blanco 2020*[33] | Brazil | 01/08/2008 | 31/12/2018 | Case series | <18y | 90 | Meningitis: prevalence, incidence, mortality |
| Brandileone 2003*[34] | Brazil | 01/01/1977 | 31/12/2000 | Cross sectional/  Surveillance | All ages | 4858 | IPD: serotypes |
| Brandileone 2021*[35] | Brazil | 01/01/2007 | 31/12/2019 | Cross sectional/  Surveillance | All ages | 11380 | IPD: serotypes |
| Caierao 2014*[36] | Brazil | 01/01/2007 | 31/12/2012 | Cross sectional | All ages | 325 | IPD: serotypes |
| Camargos 2020*[37] | Brazil | 01/01/1990 | 31/12/2017 | Cross sectional/  Surveillance | <5y | NR | Meningitis: mortality |
| Cassiolato 2019***[38] | Brazil | 01/01/2005 | 31/12/2017 | Cross sectional | All ages | 9854 | IPD: serotypes |
| Cazentini Medeiros  2017*[39] | Brazil | 01/01/1998 | 31/12/2013 | Cross sectional/  Surveillance | All ages | 796 | IPD: prevalence, serotypes |
| Christophe 2018*[40] | Brazil | 01/01/2013 | 01/06/2015 | Cross sectional | >50y | 102 | IPD: serotypes |
| da Silva 2010*[41] | Brazil | 01/01/2005 | 31/12/2008 | Case series | All ages | 168 | Meningitis: prevalence, mortality |
| dos Santos 2013*[42] | Brazil | 01/01/2006 | 30/09/2012 | Case series | All ages | 259 | IPD: incidence |
| Gomes de Oliveira  Magalhaes 2003*[43] | Brazil | 01/06/2000 | 30/05/2001 | Cross sectional/  Surveillance | <5y | 31 | IPD: serotypes |
| Gouveia 2011*[44] | Brazil | 01/12/1995 | 30/11/2005 | Case series | All ages | 548 | Meningitis: mortality, serotypes |
| Hirose 2015*[45] | Brazil | 01/01/1998 | 31/12/2011 | Non-comparative cohort | All ages | 1339 | Meningitis: incidence, mortality |
| Jarovsky 2017**[46] | Brazil | 01/01/2000 | 30/04/2017 | Case series | All ages | 561 | IPD: incidence, mortality |
| Laval 2006*[47] | Brazil | 01/05/2000 | 31/08/2001 | Cross sectional/  Surveillance | <5y | 773 | IPD: seotypes |
| Leite 2016*[48] | Brazil | 01/07/2010 | 31/12/2013 | Case series | All ages | 82 | IPD: serotypes |
| Levin 2003*[49] | Brazil | 01/07/1991 | 31/12/1994 | Cross sectional | All ages | 165 | IPD: prevalence, mortality |
| Mantese 2002*[50] | Brazil | 01/01/1987 | 31/01/2001 | Cross sectional | <18y | 415 | Meningitis: prevalence, mortality |
| Mantese 2009*[51] | Brazil | 01/04/1999 | 31/12/2008 | Case series | <5y | 142 | IPD: serotypes |
| Mendes Lages 2020*[52] | Brazil | 01/01/2005 | 31/12/2016 | Case series | <18y | 144 | IPD: incidence, serotypes |
| Menezes 2011*[53] | Brazil | 01/01/2000 | 31/12/2007 | Cross sectional/  Surveillance | All ages | 421 | Meningitis: incidence, serotypes |
| Mott 2014*[54] | Brazil | 01/01/2010 | 30/04/2012 | Cross sectional/  Surveillance | All ages | 159 | IPD: serotypes |
| Nascimento-Carvalho  2003*[55] | Brazil | 01/09/1997 | 31/05/2002 | Cross sectional/  Surveillance | <18y | 70 | IPD: serotypes |
| Neves Reis 2002*[56] | Brazil | 01/12/1995 | 30/11/1999 | Case series | All ages | 305 | Meningitis: incidence, mortality, serotypes, |
| Novaes 2011*[57] | Brazil | 01/01/2004 | 31/12/2006 | Cross sectional/  Surveillance | All ages | NR | IPD: mortality |
| Oliveira 2019*[58] | Brazil | 01/01/2005 | 31/12/2013 | Ecological study | All ages | 3963 | Meningitis: incidence |
| Pinto 2019*[59] | Brazil | 01/01/1990 | 31/12/2014 | Cross sectional | All ages | 783 | IPD: serotypes |
| Rocha Dullius 2018*[60] | Brazil | 01/01/2005 | 31/12/2016 | Cross sectional | NR | 118 | IPD: mortality, serotypes |
| Rossoni 2008*[61] | Brazil | 01/04/2001 | 30/08/2002 | Cross sectional | All ages | 436 | Meningitis: prevalence, mortality, serotypes |
| Soares dos Santo  2022*[62] | Brazil | 01/01/1996 | 31/12/2012 | Cross sectional/  Surveillance | NR | 917 | Meningitis: prevalence, mortality, serotypes |
| Veras 2007*[63] | Brazil | 01/11/2000 | 30/08/2004 | Case series | ≥18y | 79 | IPD: mortality, serotypes |
| Vieira 2007*[64] | Brazil | 01/01/1995 | 31/12/2004 | Case series | All ages | 232 | Meningitis: serotypes |
| Yoshioka 2011*[65] | Brazil | 01/01/2003 | 30/10/2008 | Cross sectional | <18y | 107 | Pneumonia: serotypes |
| Aguilera 2010*[66] | Chile | 01/01/2005 | 30/08/2006 | Case series | ≥18y | 56 | Bacteriemia: serotypes |
| Alvarado 2018*[67] | Chile | 01/01/2009 | 31/12/2015 | Ecological study | <5y | 169 | IPD: incidence, mortality, serotypes |
| Contreras 2002*[68] | Chile | 01/04/1994 | 30/05/1999 | Cross sectional | <18y | 78 | IPD: mortality, serotypes |
| Fica 2014*[69] | Chile | 01/01/2005 | 31/12/2010 | Case series | ≥18y | 59 | Pneumonia: mortality, serotypes |
| Inostroza 2007*[70] | Chile | 01/01/1994 | 31/11/2004 | Cross sectional/  Surveillance | All ages | 514 | IPD: incidence, serotypes |
| Abarca 2008*[71] | Chile | 01/05/2001 | 30/04/2002 | Cross sectional/  Surveillance | <2y | 4369 | IPD: prevalence, incidence, mortality, serotypes |
| Lagos 2008*[72] | Chile | 01/01/994 | 31/12/2007 | Cross sectional/  Surveillance | <18y | 2369 | IPD: incidence, mortality, serotypes |
| Maldonado 2007*[73] | Chile | 01/0!/2000 | 31/12/2006 | Cross sectional/  Surveillance | ≥18y | 1429 | IPD: serotypes |
| Rioseco 2004*[74] | Chile | 01/01/1997 | 31/08/2002 | Case series | ≥18y | 45 | Pneumonia: mortality |
| Rioseco 2018*[75] | Chile | 01/01/2010 | 31/12/2014 | Case series | ≥18y | 70 | Pneumonia: mortality, serotypes |
| Saldías 2011*[76] | Chile | 01/01/2002 | 31/12/2005 | Case series | ≥18y | 151 | Pneumonia: prevalence |
| Valenzuela 2014*[77] | Chile | 01/01/2007 | 31/12/2012 | Cross sectional/  Surveillance | All ages | 4829 | IPD: incidence, serotypes |
| Africano 2020*[78] | Colombia | 01/01/2012 | 31/12/2019 | Cross sectional | ≥18y | 310 | IPD: incidence, serotypes |
| Agudelo 2001*[79] | Colombia | 01/01/1994 | 31/12/2000 | Cross sectional/  Surveillance | <5y | 764 | IPD: serotypes |
| Agudelo 2002*[80] | Colombia | 01/01/1998 | 30/11/2001 | Cross sectional/  Surveillance | >5y | 343 | IPD: serotypes |
| Agudelo 2006*[81] | Colombia | 01/01/1994 | 31/12/2004 | Cross sectional/  Surveillance | All ages | 2022 | IPD: serotypes, antimicrobial susceptibility |
| Caceres 2018*[82] | Colombia | 01/01/2005 | 31/12/2015 | Ecological study | All ages | 1056 | Meningitis: prevalence |
| Calderon 2014*[83] | Colombia | 01/01/2010 | 30/06/2011 | Cross sectional | ≥18y | 60 | IPD: mortality |
| Camacho Moreno  2020*[84] | Colombia | 01/01/2008 | 31/12/2017 | Case series | <18y | 463 | IPD: mortality, serotypes |
| Farfán-Albarracín  2022*[85] | Colombia | 01/01/2008 | 31/12/2019 | Case series | <18y | 81 | Meningitis: incidence, mortality, serotypes |
| Gutierrez-Tobar 2022*[86] | Colombia | 01/01/2008 | 31/12/2019 | Cross sectional/  Surveillance | <18y | 566 | Pneumonia: prevalence, incidence, serotypes |
| Leal Castro 2022*[87] | Colombia | 01/01/2011 | 31/12/2017 | Case series | ≥18y | 169 | IPD: mortality, serotypes |
| Moreno 2004*[88] | Colombia | 01/01/2000 | 31/12/2003 | Cross sectional/  Surveillance | <5y | 190 | IPD: serotypes |
| Narváez 2021*[89] | Colombia | 01/01/2012 | 31/01/2019 | Case series | ≥18y | 310 | IPD: mortality, serotypes |
| Parra 2014*[90] | Colombia | 01/01/2005 | 31/12/2010 | Cross sectional/  Surveillance | All ages | 1775 | IPD: serotypes |
| Parra 2017*[91] | Colombia | 01/01/1994 | 31/12/2013 | Cross sectional/  Surveillance | All ages | 4991 | IPD: serotypes |
| Rojas 2016*[92] | Colombia | 01/01/2008 | 15/01/2014 | Case series | <18y | 239 | IPD: mortality |
| Severiche-Bueno  2021*[93] | Colombia | 01/01/2007 | 31/12/2017 | Cross sectional/  Surveillance | All ages | 1670 | IPD: incidence, serotypes |
| Vela 2001*[94] | Colombia | 01/02/1994 | 31/12/1999 | Cross sectional/  Surveillance | <5y | 167 | IPD: serotypes |
| Barboza 2018**[95] | Costa Rica | 01/01/2009 | 31/12/2015 | Cross sectional | <2y | 76 | Meningitis: prevalence, mortality |
| Ulloa-Gutierrez 2003*[96] | Costa Rica | 01/01/1995 | 31/12/2001 | Case series | <14y | 132 | IPD: incidence, mortality |
| Vargas-Gutierrez  2015**[97] | Costa Rica | 01/01/2006 | 31/01/2014 | Case series | <14y | 121 | Pneumonia: prevalence, serotypes |
| Batista Caluff 2017*[98] | Cuba | 01/01/2011 | 31/12/2015 | Cross sectional | <5y | 42 | IPD: mortality, serotypes |
| Dickinson Meneses  2017*[99] | Cuba | 01/01/1998 | 31/12/2015 | Prospective cohort | <5y | 483 | Meningitis: incidence, mortality |
| Fonseca Hernandez  2017*[100] | Cuba | 01/01/2014 | 31/03/2016 | Case series | <5y | 37 | IPD: mortality, serotypes |
| Morera Álvarez 2019*[101] | Cuba | 01/01/2009 | 31/12/2015 | Cross sectional | <5y | 94 | IPD: incidence, mortality, serotypes |
| Pérez 2009*[102] | Cuba | 01/01/1998 | 31/12/2007 | Case series | All ages | 4798 | Meningitis: prevalence, mortality |
| Rodríguez Cutting  2017*[103] | Cuba | 01/01/2000 | 31/12/2014 | Case series | <18y | 707 | Pneumonia: prevalence |
| Toraño Peraza 2014*[104] | Cuba | 01/01/2007 | 31/12/2012 | Case series | All ages | 237 | Meningitis: serotypes |
| Toraño Peraza 2017*[105] | Cuba | 01/01/2013 | 31/12/2015 | Case series | <18y | 141 | IPD: serotypes |
| Ahmed 2021*[106] | Dominican Republic | 01/07/2009 | 30/06/2016 | Cross sectional/  Surveillance | <14y | 342 | Pneumonia: prevalence, serotypes |
| Jonnalagadda 2017*[107] | Ecuador | 01/02/2008 | 20/04/2010 | Cross sectional | <5y | 403 | Pneumonia: prevalence |
| Juliao 2021*[108] | Ecuador | 02/01/2005 | 31/12/2017 | Cross sectional/  Surveillance | <5y | NR | IPD: incidence, mortality |
| Elenga 2015*[109] | French Guiana | 01/01/2000 | 31/12/2010 | Case series | <18y | 60 | Meningitis: mortality, serotypes |
| Gaensbauer 2016*[110] | Guatemala | 01/10/1996 | 31/12/2007 | Case series | <5y | 452 | IPD: mortality, serotypes |
| Melgar 2019**[111] | Guatemala | 01/03/2016 | 31/03/2019 | Case series | All ages | 1119 | IPD: prevalence, mortality, serotypes  Meningitis: mortality |
| Trotman 2009*[112] | Jamaica | 01/01/1995 | 31/12/1999 | Case series | <5y | 25 | Meningitis: mortality |
| Willis 2018*[113] | Jamaica | 01/01/2008 | 31/12/2009 | Prospective cohort | All ages | 350 | IPD: serotypes |
| Willis 2019*[114] | Jamaica | 01/01/2008 | 31/12/2009 | Case series | All ages | 94 | IPD: mortality |
| Alves Cardozo 2008*[115] | LAC | 01/07/1998 | 31/12/2002 | Cross sectional | <5y | 240 | Pneumonia: mortality, serotypes |
| Andrade 2012*[116]] | LAC | 01/01/2007 | 31/12/2009 | Cross sectional/  Surveillance | <5y | 31571 | IPD: prevalence, serotypes |
| Hortal 2000b*[117] | LAC | 01/01/1993 | 20/09/1999 | Cross sectional/  Surveillance | <5y | 3393 | Pneumonia: mortality, serotypes |
| Moreno 2020*[118] | LAC | 01/01/2000 | 31/12/2015 | Cross sectional/  Surveillance | All ages | 185 | IPD: serotypes |
| Peltola 2021*[119] | LAC | 01/01/1995 | 31/12/2003 | Cross sectional | <18y | 654 | Meningitis: prevalence, mortality |
| Zemlickova 2005*[120] | LAC | 01/01/2000 | 31/12/2002 | Cross sectional/  Surveillance | <5y | 185 | IPD: serotypes |
| Arredondo-García  2011*[121] | Mexico | 01/02/2002 | 31/12/2005 | Cross sectional | <18y | 150 | IPD: mortality, serotypes |
| Chacon Cruz 2017**[122] | Mexico | 01/10/2005 | 31/03/2017 | Case series | <18y | 57 | IPD: mortality, serotypes |
| Chacon Cruz 2019*[123] | Mexico | 01/10/2005 | 31/01/2018 | Case series | <18y | 64 | Pneumonia: serotypes |
| Echaniz-Aviles 2015*[124] | Mexico | 01/01/1993 | 31/12/2012 | Cross sectional/  Surveillance | <5y | 1346 | IPD: prevalence, serotypes |
| Echaniz-Aviles 2019*[125] | Mexico | 01/01/2000 | 30/09/2015 | Case series | ≥18y | 96 | IPD: mortality, serotypes |
| Franco-Paredes  2008*[126] | Mexico | 01/01/1993 | 31/12/2003 | Case series | <18y | 218 | Meningitis: prevalence |
| Gómez-Barreto 2010*[127] | Mexico | 01/01/1997 | 31/08/2014 | Case series | <14y | 156 | IPD: mortality, serotypes |
| Zarco Marquez 2016*[128] | Mexico | 01/01/2007 | 31/12/2015 | Case series | ≥18y | 69 | IPD: mortality, serotypes |
| De León 2011*[129] | Panama | 01/01/2010 | 30/06/2011 | Case series | <18y | 23 | IPD: mortality, serotypes |
| Aranda 2014*[130] | Paraguay | 01/01/1993 | 30/06/2006 | Cross sectional | <18y | 394 | Meningitis: mortality, serotypes |
| Leon 2020*[131] | Paraguay | 01/01/2010 | 31/12/2018 | Cross sectional/  Surveillance | All ages | 793 | IPD: serotypes |
| Lovera 2011*[132] | Paraguay | 01/01/200 | 30/04/2010 | Case series | <18y | 46 | Meningitis: mortality, serotypes |
| Sanabria 2009*[133] | Paraguay | 01/01/2002 | 30/08/2007 | Cross sectional | <18y | 78 | IPD: mortality, serotypes |
| Castro 2017*[134] | Peru | 01/06/2009 | 30/06/2011 | Case series | ≥18y | 43 | IPD: mortality, serotypes |
| Castillo-Tokumori  2018**[135] | Peru | 01/11/2016 | 28/0272018 | Case series | <5y | 45 | IPD: prevalence, serotypes |
| Hawkins 2017*[136] | Peru | 01/01/2006 | 31/12/2011 | Cross sectional/  Surveillance | NR | 212 | IPD: serotypes |
| Luna-Muschi 2019*[137] | Peru | 01/01/2006 | 31/12/2011 | Cross sectional/  Surveillance | <18y | 159 | IPD: mortality, incidence, serotypes |
| Morales de Santa  Gadea 2003*[138] | Peru | 01/10/2000 | 31/12/2001 | Cross sectional | <5y | 1283 | IPD: mortality, serotypes |
| Rivera-Matos 2005*[139] | Puerto Rico | 01/01/2001 | 31/12/2001 | Cross sectional/  Surveillance | All ages | 192 | IPD: mortality, serotypes |
| Nurse Lucas 2016*[140] | Trinidad and Tobago | 01/01/1997 | 31/12/2013 | Cross sectional/  Surveillance | All ages | 83 | IPD: serotypes |
| Assandri 2015*[141] | Uruguay | 01/01/2001 | 31/12/2013 | Case series | <1y | 25 | IPD: mortality, serotypes |
| Camou 2003*[142] | Uruguay | 01/01/1994 | 31/12/2001 | Cross sectional/  Surveillance | <5y | 506 | IPD: mortality, serotypes |
| Cardinal-Fernández  2013*[143] | Uruguay | 01/01/2008 | 31/10/2010 | Prospective cohort | >18y | 192 | IPD: mortality, serotypes |
| Ferrari Castilla 2007*[144] | Uruguay | 01/01/1998 | 31/12/2004 | Cross sectional/  Surveillance | <18y | 512 | Pneumonia: serotypes |
| Gabarrot 2014*[145] | Uruguay | 01/01/2003 | 31/12/2012 | Retrospective cohort | All ages | 1887 | IPD: incidence, serotypes |
| Hortal 2000a*[146] | Uruguay | 01/01/1987 | 31/12/1997 | Cross sectional/  Surveillance | >5y | 228 | IPD: mortality, serotypes |
| Hortal 2007*[147] | Uruguay | 01/01/2001 | 31/05/2004 | Cross sectional/  Surveillance | <5y | 2034 | Pneumonia: prevalence, serotypes |
| Hortal 2008*[148] | Uruguay | 01/06/2000 | 31/12/2004 | Case series | <14y | 410 | Pneumonia: mortality, serotypes |
| Hortal 2014*[149] | Uruguay | 01/01/2009 | 31/12/2012 | Cross sectional/  Surveillance | <14y | 3677 | Pneumonia: prevalence, serotypes |
| Machado 2014*[150] | Uruguay | 01/01/2010 | 31/12/2010 | Case series | <18y | 43 | Pneumonia: mortality, serotypes |
| Machado 2020*[151] | Uruguay | 01/01/2009 | 31/12/2018 | Case series | <14y | 197 | Pneumonia: prevalence, mortality, serotypes |
| Pírez 2001*[152] | Uruguay | 01/09/1997 | 31/08/1998 | Case series | <5y | 1082 | Pneumonia: prevalence, mortality |
| Pírez 2014*[153] | Uruguay | 01/01/2003 | 31/12/2012 | Non-comparative cohort | <18y | 630 | Pneumonia: incidence |
| Pírez 2017*[154] | Uruguay | 01/01/2005 | 31/12/2014 | Case series | <18y | 52 | Meningitis: prevalence, mortality, serotypes |
| Pírez García 2008*[155] | Uruguay | 01/01/1998 | 31/12/2005 | Case series | <2y | 192 | Pneumonia: mortality, serotypes |

NR: Not Reported

*Full text; **Abstract/Poster

#IPD: Invasive Pneumococcal disease

**S3 Table 3. Meta-analysis density incidence of IPD by age**

| **Study** | **Events** | **Person-time** | **Incidence Rate**  **(per 100.000 person/years)** | **I^2^** |
| --- | --- | --- | --- | --- |
| **Age 0-5** |  |  |  |  |
| Alvarado 2009 Chile <5y | 26 | 189781 | 13,7 [8,43;18,96] |  |
| Alvarado 2010 Chile <5y | 41 | 193396,2 | 21,2 [14,71; 27,68] |  |
| Alvarado 2011 Chile <5y | 22 | 194690,3 | 11,3 [6,57; 16,02] |  |
| Alvarado 2012 Chile <5y | 22 | 196428,6 | 11,2 [6,51; 15,88] |  |
| Alvarado 2013 Chile <5y | 20 | 196078,4 | 10,2 [5,72; 14,67] |  |
| Alvarado 2014 Chile <5y | 16 | 195122 | 8,2 [4,18; 12,21] |  |
| Alvarado 2015 Chile <5y | 26 | 195488,7 | 13,3 [8,18; 18,41] |  |
| Valenzuela 2014 Chile <1y 2007 | 139 | 247771,8 | 56,1 [46,77; 65,42] |  |
| Valenzuela 2014 Chile 1-2y 2007 | 104 | 247619 | 42 [33,92; 50,07] |  |
| Valenzuela 2014 Chile 2-5y 2007 | 81 | 750000 | 10,8 [8,44; 13,15] |  |
| Valenzuela 2014 Chile <1y 2008 | 114 | 250000 | 45,6 [37,22; 53,97] |  |
| Valenzuela 2014 Chile 1-2y 2008 | 120 | 248962,7 | 48,2 [39,57; 56,82] |  |
| Valenzuela 2014 Chile 2-5y 2008 | 93 | 744000 | 12,5 [9,95; 15,04] |  |
| Valenzuela 2014 Chile <1y 2009 | 121 | 251037,3 | 48,2 [39,61; 56,78] |  |
| Valenzuela 2014 Chile 1-2y 2009 | 103 | 250000 | 41,2 [33,24; 49,15] |  |
| Valenzuela 2014 Chile 2-5y 2009 | 83 | 747747,7 | 11,1 [8,71; 13,48] |  |
| Valenzuela 2014 Chile <1y 2010 | 92 | 252054,8 | 36,5 [29,04; 43,95] |  |
| Valenzuela 2014 Chile 1-2y 2010 | 100 | 250626,6 | 39,9 [32,07; 47,72] |  |
| Valenzuela 2014 Chile 2-5y 2010 | 81 | 750000 | 10,8 [8,44; 13,15] |  |
| Valenzuela 2014 Chile <1y 2011 | 81 | 252336,4 | 32,1 [25,10; 39,09] |  |
| Valenzuela 2014 Chile 1-2y 2011 | 88 | 250712,3 | 35,1 [27,76; 42,43] |  |
| Valenzuela 2014 Chile 2-5y 2011 | 68 | 755555,6 | 9 [6,86; 11,13] |  |
| Valenzuela 2014 Chile <1y 2012 | 41 | 251533,7 | 16,3 [11,31; 21,28] |  |
| Valenzuela 2014 Chile 1-2y 2012 | 50 | 251256,3 | 19,9 [14,38; 25,41] |  |
| Valenzuela 2014 Chile 2-5y 2012 | 80 | 747663,6 | 10,7 [8,35; 13,04] |  |
| Gabarrot 2003-2007 Uruguay <2y | 67,2 | 97816,6 | 68,7 [52,27; 85,12] |  |
| Gabarrot 2003-2007 Uruguay 2-5y | 36 | 151133,5 | 23,82 [16,03; 31,60] |  |
| Gabarrot 2009-2012 Uruguay <2y | 22,25 | 95698,9 | 23,25 [13,58; 32,91] |  |
| Gabarrot 2009-2012 Uruguay 2-5y | 15,75 | 144098,8 | 10,93 [5,53; 16,32] |  |
| Luna-Muschi 2019 Peru 1-2y | 12 | 225500 | 5,1 [2,15; 8,05] |  |
| **Random effects model** |  |  | **24,29 [18,41;30,18]** | **95%** |
| **Age 6-64** |  |  |  |  |
| Valenzuela 2014 Chile 6-64y 2007 | 295 | 14047619 | 2,1 [1,86; 2,33] |  |
| Valenzuela 2014 6-64y 2008 | 375 | 13888888,9 | 2,7 [2,42; 2,97] |  |
| Valenzuela 2014 Chile 6-64y 2009 | 397 | 13689655,2 | 2,9 [2,61; 3,18 ] |  |
| Valenzuela 2014 Chile 6-64y 2010 | 303 | 14428571,4 | 2,1 [1,86; 2,33] |  |
| Valenzuela 2014 Chile 6-64y 2011 | 305 | 14523809,5 | 2,1 [1,86; 2,33] |  |
| Valenzuela 2014 Chile 6-64y 2012 | 321 | 15285714,3 | 2,1 [1,87; 2,32] |  |
| **Random effects model** |  |  | **2,33 [2,04; 2,61]** | **86%** |
| **Age 65-100** |  |  |  |  |
| Valenzuela 2014 Chile ≥65y 2007 | 149 | 1392523,4 | 10,7 [8,98; 12,41] |  |
| Valenzuela 2014 ≥65y 2008 | 123 | 1464285,7 | 8,4 [6,91; 9,88 ] |  |
| Valenzuela 2014 Chile ≥65y 2009 | 136 | 1528089,9 | 8,9 [7,40; 10,39 ] |  |
| Valenzuela 2014 Chile ≥65y 2010 | 141 | 1566666,7 | 9 [7,51; 10,48] |  |
| Valenzuela 2014 Chile ≥65y 2011 | 113 | 1614285,7 | 7 [5,70; 8,29] |  |
| Valenzuela 2014 Chile ≥65y 2012 | 156 | 1677419,4 | 9,3 [7,84; 10,75] |  |
| **Random effects model** |  |  | **8,81 [7,85; 9,78]** | **61%** |

**S3 Table 4. Meta-analysis density incidence of meningitis in children under 5 years of age**

| **Study** | **Event** | **Person-time** | **Rate**  **(per 100.000 person/years)** | **I^2^** |
| --- | --- | --- | --- | --- |
| **Age 0-5** |  |  |  |  |
| Dickinson-Meneses 2017 Cuba 1998-2015 <6y | 26,8 | 789216 | 3,39 [ 2,11; 4,68] |  |
| Grenón 2014 Argentina 1994-2009 1-2y | 1,7 | 13281 | 12,80 [ 0,00; 32,04] |  |
| Grenón 2014 Argentina 1994-2009 2-4y | 1,2 | 13187 | 9,09 [ 0,00; 25,38] |  |
| Hirose 2015 Brazil 1998-2009 <1y | 26,1 | 175758 | 14,85 [ 9,15; 20,54] |  |
| Hirose 2015 Brazil 2010-2011 <1y | 8 | 144665 | 5,53 [ 1,70; 9,36] |  |
| Neves-Reis 2002 Brazil <5y | 24,7 | 100000 | 24,7 [14.95; 34.44] |  |
| Ulloa-Gutierrez 2003 Costa-Rica <2y | 4,8 | 235294 | 2,04 [ 0.21; 3.86] |  |
| **Random effects model** |  |  | **9,28 [3,07; 15,49]** | **84,3%** |

**S3 Table 5. Risk of bias assessment for cohort and cross sectional studies**

| **Author and year of**  **publication** | **Evaluation *** | | | | | | | | | | | | | | |
| --- | --- | --- | --- | --- | --- | --- | --- | --- | --- | --- | --- | --- | --- | --- | --- |
|  | **1** | **2** | **3** | **4** | **5** | **6** | **7** | **8** | **9** | **10** | **11** | **12** | **13** | **14** | **Final** |
| **Abarca 2008 [**[71] | Yes | Yes | CD | Yes | No | NA | NA | No | Yes | No | Yes | NA | NA | NA | **Fair** |
| **Abate 2014** [28] | Yes | Yes | Yes | Yes | No | NA | NA | No | Yes | No | Yes | No | NA | No | **Fair** |
| **Africano 2020 [**[78] | Yes | Yes | CD | Yes | No | NA | NA | No | Yes | No | Yes | No | NA | NA | **Fair** |
| **Agudelo 2001 [**[79]**]** | Yes | Yes | Yes | Yes | No | NA | NA | No | Yes | No | Yes | NA | NA | NA | **Good** |
| **Agudelo 2002 [**[80]**]** | Yes | Yes | Yes | Yes | No | NA | NA | No | Yes | No | Yes | NA | NA | NA | **Good** |
| **Agudelo 2006** [81]**]** | Yes | Yes | Yes | Yes | No | NA | NA | No | Yes | No | Yes | NA | NA | NA | **Good** |
| **Ahmed 2021 [**[106]**]** | Yes | Yes | CD | Yes | No | NA | NA | No | Yes | No | Yes | NA | NA | NA | **Fair** |
| **Altclas 2004** [2]**]** | Yes | Yes | CD | Yes | No | NA | NA | No | Yes | No | Yes | No | NA | No | **Poor** |
| **Alvarado 2018** [67]**]** | NA | NA | NA | NA | NA | NA | NA | NA | NA | NA | NA | NA | NA | NA | NA |
| **Alves Cardozo 2008 [**[115] | Yes | Yes | CD | Yes | Yes | NA | NA | Yes | Yes | NA | Yes | No | NA | Yes | **Fair** |
| **Andrade 2012 [**[116] | Yes | Yes | Yes | Yes | No | No | NA | No | Yes | No | Yes | No | NA | NA | **Good** |
| **Aranda 2014 [**[130]**]** | Yes | Yes | Yes | Yes | No | NA | NA | NA | Yes | NA | Yes | No | NA | No | **Fair** |
| **Arredondo-García 2011 [**1[121] | Yes | Yes | CD | Yes | No | NA | NA | No | Yes | No | Yes | No | NA | NA | **Fair** |
| **Azevedo 2016** [28] | Yes | Yes | CD | Yes | No | Yes | Yes | No | Yes | NA | Yes | No | NA | NA | **Fair** |
| **Bakir 2003** [3] | Yes | Yes | CD | Yes | No | NA | NA | No | Yes | NA | Yes | No | NA | No | **Poor** |
| **Barboza 2002** [4]**]** | Yes | Yes | Yes | Yes | No | NA | NA | No | Yes | No | Yes | No | NA | NA | **Good** |
| **Barboza 2018 [**[95]**]** | Yes | Yes | No | Yes | No | NA | NA | No | Yes | No | Yes | NA | NA | NA | **Fair** |
| **Barroso 2012 [**[29]**]** | Yes | Yes | No | Yes | No | No | NA | NA | Yes | No | Yes | NA | NA | NA | **Fair** |
| **Batista Caluff 2017 [**[98]**]** | Yes | Yes | Yes | Yes | No | NA | NA | No | Yes | No | Yes | No | NA | NA | **Good** |
| **Benitez 2017 [**[5] | Yes | Yes | Yes | Yes | No | NA | NA | No | Yes | No | Yes | No | NA | NA | **Good** |
| **Berberian 2014 [**[6] | Yes | Yes | No | Yes | No | Yes | Yes | No | Yes | No | Yes | No | No | No | **Good** |
| **Brandileone 2003** [61] | Yes | Yes | Yes | Yes | No | NA | NA | Yes | Yes | No | Yes | NA | NA | NA | **Good** |
| **Brandileone 2021** [35] | Yes | Yes | Yes | CD | No | No | NA | No | Yes | No | Yes | No | NA | NA | **Good** |
| **Cáceres 2018 [**[82] | NA | NA | NA | NA | NA | NA | NA | NA | NA | NA | NA | NA | NA | NA | NA |
| **Caierao 2014 [**[36] | Yes | Yes | CD | Yes | No | NA | NA | No | Yes | No | Yes | No | NA | NA | **Fair** |
| **Calderon 2014 [**[83] | Yes | Yes | CD | Yes | No | NA | NA | No | Yes | NA | Yes | No | NA | No | **Poor** |
| **Camargos 2020 [**[37]**]** | Yes | No | Yes | Yes | No | No | Yes | No | Yes | Yes | Yes | No | CD | No | **Poor** |
| **Camou 2003 [**[142] | Yes | Yes | Yes | Yes | No | No | NA | No | Yes | No | Yes | No | NA | NA | **Good** |
| **Cardinal-Fernández 2013 [**[143] | Yes | Yes | Yes | Yes | No | Yes | Yes | No | Yes | No | Yes | No | CD | No | **Fair** |
| **Cassiolato 2019** [65] | Yes | Yes | Yes | Yes | No | NA | NA | No | Yes | No | Yes | No | NA | NA | **Good** |
| **Cazentini Medeiros 2017 [**[39] | Yes | Yes | CD | CD | No | NA | NA | No | Yes | No | Yes | NA | NA | NA | **Fair** |
| **Christophe 2018 [**[40] | Yes | Yes | CD | Yes | No | NA | NA | No | Yes | No | Yes | No | NA | NA | **Fair** |
| **Contreras 2002 [**[68]**]** | Yes | Yes | No | Yes | No | Yes | Yes | No | Yes | No | Yes | No | No | No | **Poor** |
| **Dickinson Meneses 2017 [**[99] | Yes | Yes | CD | Yes | No | Yes | Yes | Yes | Yes | No | Yes | No | Yes | No | **Poor** |
| **Echaniz-Aviles 2015 [**1[124]**]** | Yes | Yes | CD | Yes | Yes | Yes | NA | No | Yes | NA | Yes | No | NA | Yes | **Fair** |
| **Ferrari Castilla 2007 [**[144] | Yes | Yes | CD | Yes | No | NA | NA | No | Yes | NA | Yes | NA | NA | NA | **Fair** |
| **Gabarrot 2014** [145] | Yes | Yes | No | Yes | No | Yes | Yes | Yes | Yes | No | Yes | No | Yes | NA | **Fair** |
| **Gagetti 2017** [36] | Yes | Yes | Yes | Yes | No | NA | NA | NA | Yes | NA | Yes | NA | NA | NA | **Good** |
| **Gagetti 2021** [10]**]** | Yes | Yes | Yes | Yes | Yes | NA | NA | NA | Yes | No | Yes | NA | NA | NA | **Fair** |
| **Gentile 2018a** [12]**]** | Yes | Yes | Yes | Yes | No | No | NA | Yes | Yes | NA | Yes | No | NA | No | **Fair** |
| **Gentile 2018b [**[13]**]** | Yes | Yes | No | Yes | No | No | No | No | Yes | No | Yes | No | No | No | **Poor** |
| **Gomes de Oliveira Magalhaes**  **2003 [**[43] | Yes | Yes | CD | Yes | No | No | NA | Yes | Yes | NA | Yes | No | NA | No | **Fair** |
| **Grenón 2005** [41] | Yes | Yes | CD | Yes | No | NA | NA | Yes | Yes | No | Yes | No | NA | NA | **Fair** |
| **Gutierrez-Tobar 2022 [**[86] | Yes | Yes | NA | Yes | No | NA | NA | NA | Yes | No | Yes | NA | NA | NA | **Good** |
| **Hawkins 2017** [136]**]** | Yes | No | Yes | Yes | No | NA | NA | No | Yes | No | Yes | No | NA | NA | **Fair** |
| **Hirose 2015** [45] | Yes | Yes | CD | Yes | No | Yes | Yes | Yes | Yes | No | Yes | No | Yes | NA | **Fair** |
| **Hortal 2000a [**[146] | Yes | Yes | Yes | Yes | No | NA | NA | No | Yes | No | Yes | NA | NA | NA | **Fair** |
| **Hortal 2000b [**[117] | Yes | Yes | Yes | Yes | No | NA | NA | Yes | Yes | No | Yes | No | NA | NA | **Good** |
| **Hortal 2007 [**[147] | Yes | Yes | Yes | Yes | No | NA | NA | Yes | Yes | No | Yes | No | NA | NA | **Good** |
| **Hortal 2014 [**[149]**]** | Yes | Yes | Yes | Yes | No | NA | NA | Yes | Yes | No | Yes | No | NA | NA | **Good** |
| **Inostroza 2007 [**[70]**]** | Yes | Yes | Yes | Yes | No | NA | NA | Yes | Yes | No | Yes | No | NA | NA | **Good** |
| **Jonnalagadda 2017** [107] | Yes | Yes | CD | Yes | No | No | NA | No | Yes | No | Yes | No | NA | NA | **Fair** |
| **Juliao 2021 [**[108] | Yes | Yes | Yes | Yes | Yes | No | Yes | No | Yes | No | Yes | No | NA | Yes | **Fair** |
| **Lagos 2008 [**[72]**]** | Yes | Yes | CD | Yes | Yes | Yes | Yes | No | Yes | Yes | Yes | No | CD | Yes | **Good** |
| **Laval 2006 [**[47]**]** | Yes | Yes | CD | Yes | Yes | NA | NA | No | Yes | No | Yes | NA | NA | NA | **Fair** |
| **León 2020 [**[131] | Yes | Yes | CD | Yes | No | No | NA | No | Yes | NA | Yes | No | NA | No | **Fair** |
| **Levin 2003 [**[49]**]** | Yes | Yes | CD | Yes | No | Yes | NA | No | Yes | NA | Yes | No | NA | No | **Fair** |
| **Lopez 2018 [**[16] | Yes | Yes | CD | Yes | No | No | Yes | No | Yes | No | Yes | No | CD | No | **Poor** |
| **Luna-Muschi 2019** [137]**]** | Yes | Yes | Yes | Yes | No | NA | NA | No | Yes | No | Yes | NA | NA | NA | **Good** |
| **Maldonado 2007 [**[73] | Yes | Yes | Yes | Yes | No | Yes | Yes | No | Yes | No | Yes | No | No | No | **Fair** |
| **Mantese 2002***[50]**]** | Yes | Yes | CD | Yes | No | No | NA | No | Yes | NA | Yes | No | NA | No | **Fair** |
| **Mathurin 2008** [44] | Yes | Yes | CD | Yes | No | Yes | Yes | No | Yes | No | Yes | No | No | No | **Poor** |
| **Menezes 2011[**[53] | Yes | Yes | Yes | Yes | No | No | NA | No | Yes | NA | Yes | NA | NA | NA | **Fair** |
| **Morales de Santa**  **Gadea 2003 [**[138]**]** | Yes | Yes | Yes | Yes | No | NA | NA | Yes | Yes | No | Yes | No | NA | NA | **Good** |
| **Moreno 2004 [**[88]**]** | Yes | Yes | CD | Yes | No | NA | NA | No | Yes | No | Yes | NA | NA | NA | **Fair** |
| **Moreno 2020* [**[118] | Yes | Yes | CD | Yes | No | NA | NA | No | Yes | No | Yes | NA | NA | NA | **Fair** |
| **Morera Álvarez 2019 [**[101] | Yes | Yes | No | Yes | No | NA | NA | No | Yes | NA | Yes | No | NA | NA | **Fair** |
| **Mott 2014 [**[54] | Yes | Yes | CD | Yes | No | NA | NA | No | Yes | No | Yes | NA | NA | NA | **Fair** |
| **Nascimento-Carvalho 2003 [**[55] | Yes | Yes | CD | Yes | Yes | Yes | NA | No | Yes | No | Yes | No | NA | NA | **Fair** |
| **Novaes 2011[**[57] | Yes | Yes | Yes | Yes | No | NA | NA | No | Yes | NA | Yes | No | NA | No | **Fair** |
| **Nurse Lucas 2016[**[140] | Yes | Yes | CD | CD | No | No | NA | No | No | No | Yes | No | NA | NA | **Fair** |
| **Oliveira 2019 [**[58]**]** | NA | NA | NA | NA | NA | NA | NA | NA | NA | NA | NA | NA | NA | NA | NA |
| **Paganini 2001** [46] | Yes | Yes | CD | Yes | No | No | NA | Yes | Yes | NA | Yes | No | NA | No | **Fair** |
| **Palma 2011**[20] | Yes | Yes | CD | Yes | No | No | NA | No | Yes | NA | Yes | No | NA | No | **Good** |
| **Paniagua 2007 [**[21] | Yes | Yes | Yes | Yes | No | No | NA | No | Yes | No | Yes | No | NA | NA | **Good** |
| **Parra 2014 [**[90] | Yes | Yes | CD | Yes | No | NA | NA | No | Yes | No | Yes | NA | NA | NA | **Fair** |
| **Parra 2017 [**[91] | Yes | Yes | CD | Yes | No | NA | NA | No | Yes | No | Yes | NA | NA | NA | **Fair** |
| **Peltola 2021 [**[119] | Yes | Yes | CD | Yes | No | NA | NA | No | Yes | NA | Yes | No | NA | Yes | **Fair** |
| **Pinto 2019 [**[59]**]** | Yes | Yes | CD | Yes | No | No | NA | No | Yes | NA | Yes | No | NA | No | **Fair** |
| **Pírez 2014*[**[153]**]** | Yes | Yes | CD | Yes | No | Yes | Yes | Yes | Yes | No | Yes | No | CD | NA | **Fair** |
| **Rivera-Matos 2005 [**[139]**]** | Yes | Yes | CD | Yes | No | NA | NA | No | Yes | No | Yes | NA | NA | NA | **Fair** |
| **Rocha Dullius 2018 [**[60]**]** | Yes | Yes | CD | Yes | No | NA | NA | NA | Yes | No | Yes | No | NA | NA | **Fair** |
| **Rossoni 2008 [**[61] | Yes | Yes | CD | Yes | No | NA | NA | Yes | Yes | No | Yes | No | NA | NA | **Fair** |
| **Ruvinsky 2010** [51] | Yes | Yes | Yes | Yes | No | NA | NA | No | Yes | No | Yes | NA | NA | NA | **Good** |
| **Sanabria 2009 [**[133] | Yes | Yes | CD | Yes | No | Yes | NA | No | Yes | NA | Yes | No | NA | No | **Poor** |
| **Severiche-Bueno 2021** [93] | Yes | Yes | Yes | Yes | No | NA | NA | No | Yes | No | Yes | NA | NA | NA | **Fair** |
| **Soares dos Santos 2022** [62]**]** | Yes | Yes | Yes | Yes | No | NA | NA | No | Yes | No | Yes | NA | NA | NA | **Good** |
| **Tregnaghi 2006** [25] | Yes | Yes | CD | Yes | No | NA | NA | No | Yes | No | Yes | NA | NA | NA | **Fair** |
| **Valenzuela 2014** [77] | Yes | Yes | CD | Yes | No | NA | NA | No | Yes | No | Yes | NA | NA | NA | **Fair** |
| **Vela 2001** [94] | Yes | Yes | Yes | Yes | No | NA | NA | NO | Yes | No | Yes | NA | NA | NA | **Good** |
| **Willis 2018** [113] | Yes | Yes | Yes | Yes | No | Yes | Yes | No | Yes | No | Yes | No | CD | No | **Fair** |
| **Yoshioka 2011** [65]**]** | Yes | Yes | CD | Yes | No | Yes | NA | Yes | Yes | NA | Yes | No | NA | No | **Fair** |
| **Zemlickova 2005** [120]**]** | Yes | Yes | CD | Yes | No | Yes | CD | Yes | Yes | No | Yes | No | No | No | **Fair** |
| **Zintgraff 2020** [66] | Yes | Yes | CD | Yes | No | Yes | No | Yes | Yes | No | Yes | No | CD | No | **Fair** |

* **NA**: Not applicable, **CD**: Cannot be determined

1. Was the study question or research objective clearly specified?
2. Was the study population clearly specified and defined?
3. Did at least 50% of eligible subjects take part?
4. Were all subjects screened or recruited from the same population or from similar populations (including the same period of time)? Were inclusion and exclusion criteria to take part in the study pre-specified and applied consistently to all participants?
5. Was rationale for sample size, power description or variance and effect estimations provided?
6. For analysis of this study, were the exposures of interest measured before results?
7. Was the follow-up period enough for one to reasonably expect to observe an association between exposure and result, if any?
8. For exposures that may vary in terms of amount or level, did the study examine different levels of exposure relative to the result (e.g. categories of exposure or exposure measured as a continuous variable)?
9. Were measures of exposure (independent variables) clearly defined, valid, reliable and consistently implemented for all study participants?
10. Were exposures evaluated more than once over time?
11. Were measures of results (dependent variables) clearly defined, valid, reliable and consistently implemented for all study participants?
12. Were results raters blinded to participants’ exposure?
13. Were lost to follow-up 20% or less after the study startup?
14. Were potential confounding variables key due to their impact on the exposure(s)-result(s) ratio measured and statistically adjusted?

**S3 Table 6. Risk of bias assessment for case series studies**

| **Author and year of publication** | **Evaluation *** | | | | | | | | | |
| --- | --- | --- | --- | --- | --- | --- | --- | --- | --- | --- |
|  | **1** | **2** | **3** | **4** | **5** | **6** | **7** | **8** | **9** | **Final** |
| **Aguilera 2010** [66] | Yes | Yes | CD | Yes | Yes | Yes | Yes | No | Yes | **Good** |
| **Alvares 2011 [**[27]**]** | Yes | No | CD | Yes | Yes | Yes | Yes | No | Yes | **Fair** |
| **Assandri 2015 [**[141]**]** | Yes | Yes | Yes | Yes | Yes | Yes | Yes | No | Yes | **Good** |
| **Berezin 2002 [**[30] | Yes | Yes | CD | Yes | Yes | Yes | Yes | No | Yes | **Good** |
| **Berezin 2007** [31] | Yes | No | CD | Yes | Yes | Yes | Yes | No | Yes | **Fair** |
| **Berezin 2020 [**[32] | Yes | Yes | CD | Yes | Yes | Yes | Yes | No | Yes | **Good** |
| **Blanco 2020 [**[33]**]** | Yes | No | CD | Yes | Yes | Yes | Yes | No | Yes | **Fair** |
| **Camacho Moreno 2020** [111] | Yes | Yes | CD | Yes | Yes | Yes | Yes | No | Yes | **Good** |
| **Castillo-Tokumori 2018 [**[135]**]** | Yes | No | CD | Yes | Yes | Yes | Yes | No | Yes | **Fair** |
| **Castro 2017** [134]**]** | Yes | Yes | CD | Yes | Yes | Yes | Yes | Yes | Yes | **Good** |
| **Chacon Cruz 2017 [**[122] | Yes | Yes | CD | Yes | Yes | Yes | Yes | No | Yes | **Fair** |
| **Chacon Cruz 2019 [**[123] | Yes | Yes | Yes | Yes | Yes | Yes | Yes | No | Yes | **Good** |
| **Corbacho-Re 2020 [**[7] | Yes | Yes | CD | CD | Yes | Yes | Yes | Yes | Yes | **Fair** |
| **da Silva 2010** [68] | Yes | Yes | CD | Yes | Yes | Yes | Yes | No | Yes | **Good** |
| **De León 2011 [**[129]**]** | Yes | No | Yes | Yes | Yes | Yes | Yes | No | No | **Fair** |
| **dos Santos 2013 [**[42] | Yes | No | CD | Yes | Yes | Yes | Yes | No | Yes | **Fair** |
| **Echaniz-Aviles 2019 [**[125] | Yes | Yes | CD | Yes | Yes | Yes | Yes | Yes | Yes | **Good** |
| **Elenga 2015 [**[109] | Yes | Yes | CD | Yes | Yes | Yes | Yes | No | Yes | **Fair** |
| **Farfán-Albarracín 2022 [**[85] | Yes | Yes | CD | Yes | Yes | Yes | Yes | Yes | Yes | **Good** |
| **Fica 2014 [**[69] | Yes | Yes | Yes | Yes | Yes | Yes | Yes | Yes | Yes | **Good** |
| **Fonaroff 2014** [35] | Yes | Yes | CD | Yes | Yes | Yes | Yes | Yes | Yes | **Good** |
| **Fonseca Hernandez 2017 [**[100] | Yes | No | CD | Yes | Yes | Yes | Yes | No | Yes | **Fair** |
| **Franco-Paredes 2008 [**[126]**]** | Yes | Yes | CD | Yes | Yes | Yes | Yes | Yes | Yes | **Good** |
| **Gaensbauer 2016 [**[110] | Yes | Yes | Yes | Yes | Yes | Yes | Yes | No | Yes | **Good** |
| **Gentile 2003** [38] | Yes | Yes | CD | Yes | Yes | Yes | Yes | Yes | Yes | **Good** |
| **Gómez-Barreto 2010 [**[127] | Yes | Yes | CD | Yes | Yes | Yes | Yes | Yes | Yes | **Good** |
| **Gouveia 2011** [44]**]** | Yes | No | Yes | Yes | Yes | Yes | Yes | Yes | Yes | **Fair** |
| **Grenon 2014** [15]**]** | Yes | Yes | Yes | Yes | Yes | Yes | Yes | Yes | Yes | **Good** |
| **Hortal 2008 [**[148]**]** | Yes | Yes | Yes | Yes | Yes | Yes | yes | No | Yes | **Good** |
| **Jarovsky 2017 [**[46]**]** | Yes | Yes | CD | Yes | Yes | Yes | Yes | No | Yes | **Good** |
| **Leal Castro 2022 [**[87] | Yes | Yes | CD | Yes | Yes | Yes | Yes | Yes | Yes | **Good** |
| **Leite 2016 [**[48] | Yes | Yes | Yes | Yes | Yes | Yes | Yes | Yes | Yes | **Good** |
| **Lovera 2011 [**[132]**]** | Yes | Yes | Yes | Yes | Yes | Yes | Yes | Yes | Yes | **Good** |
| **Machado 2014 [**[150]**]** | Yes | Yes | CD | Yes | Yes | Yes | Yes | No | Yes | **Good** |
| **Machado 2020 [**[151] | Yes | Yes | CD | Yes | Yes | Yes | Yes | Yes | Yes | **Good** |
| **Mantese 2009 [**[51] | Yes | Yes | CD | Yes | Yes | Yes | Yes | CD | Yes | **Good** |
| **Mayoral 2008 [**[18]5] | Yes | Yes | CD | Yes | Yes | Yes | NA | Yes | Yes | **Good** |
| **Melgar 2019 [**[111]**]** | Yes | No | CD | Yes | Yes | Yes | Yes | No | No | **Fair** |
| **Mendes Lages 2020 [**[52] | Yes | Yes | Yes | Yes | Yes | Yes | Yes | Yes | Yes | **Good** |
| **Narváez 2021** [89]**]** | Yes | Yes | CD | Yes | Yes | Yes | Yes | Yes | Yes | **Good** |
| **Neves Reis 2002 [**[56]**]** | No | No | Yes | CD | Yes | Yes | Yes | No | Yes | **Poor** |
| **Pérez 2009 [**[102] | Yes | Yes | CD | Yes | Yes | Yes | Yes | Yes | Yes | **Good** |
| **Perez 2014** [49] | Yes | Yes | CD | Yes | Yes | Yes | Yes | No | Yes | **Good** |
| **Pírez 2001 [**[152] | Yes | Yes | CD | Yes | Yes | Yes | Yes | Yes | Yes | **Good** |
| **Pírez 2017** [154]**]** | Yes | Yes | Yes | Yes | Yes | NA | Yes | Yes | Yes | **Fair** |
| **Pírez García 2008 [**[155] | Yes | Yes | CD | Yes | Yes | Yes | Yes | Yes | Yes | **Good** |
| **Reijtman 2011** [50] | Yes | No | CD | Yes | Yes | Yes | Yes | No | Yes | **Fair** |
| **Rioseco 2004 [**[74]**]** | Yes | Yes | CD | Yes | Yes | Yes | Yes | Yes | Yes | **Good** |
| **Rioseco 2018** [75] | Yes | Yes | CD | Yes | Yes | Yes | Yes | Yes | Yes | **Good** |
| **Rodríguez Cutting 2017 [**[103] | Yes | Yes | CD | Yes | No | Yes | Yes | No | Yes | **Fair** |
| **Rojas 2016** [92] | Yes | No | CD | Yes | Yes | Yes | Yes | No | Yes | **Fair** |
| **Saldías 2011 [**[76] | Yes | Yes | CD | Yes | Yes | Yes | Yes | Yes | Yes | **Good** |
| **Toraño.Peraza 2014 [**[104] | Yes | Yes | CD | Yes | Yes | Yes | Yes | No | Yes | **Fair** |
| **Toraño-Peraza 2017** [105]**]** | Yes | Yes | CD | Yes | Yes | Yes | Yes | No | Yes | **Fair** |
| **Trotman 2009 [**[112]**]** | Yes | Yes | Yes | CD | Yes | Yes | Yes | Yes | Yes | **Good** |
| **Ulloa-Gutierrez 2003 [**[96] | Yes | Yes | Yes | Yes | Yes | Yes | Yes | No | Yes | **Good** |
| **Vargas-Gutierrez 2015 [**[97] | No | Yes | CD | Yes | Yes | Yes | Yes | No | Yes | **Fair** |
| **Veras 2007** [90] | Yes | Yes | CD | Yes | Yes | Yes | Yes | Yes | Yes | **Good** |
| **Vieira 2007** [64] | Yes | Yes | CD | Yes | Yes | Yes | NA | No | Yes | **Fair** |
| **Zarco Marquez 2016 [**[128] | Yes | Yes | Yes | Yes | Yes | Yes | Yes | No | Yes | **Good** |
| **Willis 2019 [**[114] | Yes | Yes | Yes | Yes | Yes | Yes | Yes | Yes | Yes | **Good** |

* **NA**: Not applicable, **CD**: Cannot be determined

1. Was the study question or objective clearly specified?
2. Was the study population clearly and fully described, including case definition?
3. Were cases consecutive?
4. Were subjects comparable?
5. Was exposure clearly described?
6. Were measures of results clearly defined, valid, reliable and consistently implemented for all study participants?
7. Was the length of follow-up appropriate?
8. Were statistical methods properly described?
9. Were results properly described

References

1. Abate HJ, Falaschi A, Balbi L, García B. Diecinueve años de vigilancia de enfermedad invasiva neumocócica en un hospital pediátrico de Mendoza, Argentina^iesNineteen-years of pneumococcal invasive disease surveillance in a children’s hospital in Mendoza, Argentina^ien. Arch Argent Pediatr. 2014;112: 352–357.

2. Altclas J, Salgueira C, Di Martino A. Pneumococcal bacteremia in a single center in Argentina. Int J Infect Dis. 2004;8: 53–58.

3. Bakir J, de Gentile AS, López Holtmann G, Procopio A, Vázquez M. Perfil epidemiológico de las infecciones invasivas por Streptococcus pneumoniae: epidemiological profile^iesInvasive streptococcus pneumoniae^ien. Rev Soc Boliv Pediatr. 2003;42: 63–68.

4. Barboza AG, Ioli PL, Zamarbide I, Estrago MI, Castineiras F, De Wouters L. A study of the incidence and a descriptive analysis of adult non-tuberculous primary bacterial meningitis in a population in Argentina. Rev Neurol. 2002;35: 508–512.

5. Benitez JD, Martinez ME, Specht MH von, Gerlach E, Gonzalez CA, Grenon SL. Epidemiology and risk factors for invasive pneumococcal disease in pediatrics. Descriptive, postvaccinal study. Epidemiologia y factores de riesgo de enfermedad invasiva neumococica en pediatria Estudio descriptivo, postvacunal. Revista de ciencia y tecnologia 2017; 4–10. https://ri.conicet.gov.ar/handle/11336/177141 Accessed May 5th 2023

6. Berberian G, Pérez M G, Epelbaum C, Ceinos MDC, Lopardo H, Rosanova MT. Meningitis neumocócica: experiencia de 12 años en un hospital pediátrico, previa a la inmunización universal con vacuna conjugada^iesPneumococcal meningitis: A 12 year experience in a children’s hospital prior to the universal immunization with a conjugat. Arch Argent Pediatr. 2014;112: 332–336.

7. Corbacho-Re MF, Kilstein JG, Negro-marquinez L. THE PERFORMANCE OF SOAR SCORE AS A PREDICTOR OF SEVERITY IN ACUTE COMMUNITYACQUIRED PNEUMONIA. Rev Med Rosario. 2020;86: 11–18.

8. Fonaroff EG. Neumonía Neumocócica Bacteriémica en el adulto^ies. Rev panam infectol. 2014;16: 79–85.

9. Gagetti P, Faccone D, Reijtman V, Fossati S, Rodriguez M, Veliz O, et al. Characterization of Streptococcus pneumoniae invasive serotype 19A isolates from Argentina (1993-2014). Vaccine. 2017;35: 4548–4553.

10. Gagetti P, Lo SW, Hawkins PA, Gladstone RA, Regueira M, Faccone D, et al. Population genetic structure, serotype distribution and antibiotic resistance of streptococcus pneumoniae causing invasive disease in children in Argentina. Microbial Genomics. 2021;7: 000636.

11. Gentile JH, Sparo MD, Mercapide ME, Luna CM. Adult bacteremic pneumococcal pneumonia acquired in the community. A prospective study on 101 patients. Medicina. 2003;63: 9–14.

12. Gentile A, Bakir J, Firpo V, Casanueva EV, Ensinck G, Papucci SL, et al. PCV13 vaccination impact: A multicenter study of pneumonia in 10 pediatric hospitals in Argentina. PLoS One. 2018;13: e0199989.

13. Gentile A, Bakir J, Lucion MF, Del Valle Juarez M, Rapaport S, Areso MS. Community-acquired bacteremic pneumonia in post-pneumococcal vaccination era in a pediatric hospital. Open Forum Infectious Diseases. 2018;5: S451–S452.

14. Grenon S, Von Specht M, Corso A, Pace J, Regueira M. Distribution of serotypes and antibiotic susceptibility patterns of Streptococcus pneumoniae strains isolated from children in Misiones, Argentina. Enferm Infecc Microbiol Clin. 2005;23: 10–14.

15. Grenón SL, Salvi Grabulosa MC, Regueira MM, Fossati MS, von Specht MH. Meningitis neumocócica en niños menores de 15 años: Dieciséis años de vigilancia epidemiológica en Misiones, Argentina^iesPneumococcal meningitis in children under 15 years of age in Misiones (Argentina): Sixteen year’s epidemiological surveillance^ien. Rev Argent Microbiol. 2014;46: 14–23.

16. Lopez PL, Torrente FM, Ciapponi A, Lischinsky AG, Cetkovich-Bakmas M, Rojas JI, et al. Cognitive-behavioural interventions for attention deficit hyperactivity disorder (ADHD) in adults. Cochrane Database Syst Rev. 2018;3: CD010840.

17. Mathurin S, Jaimet C, Aguero A, Moro J, del Pino A, Arosio A, et al. Prospective observational study of pneumococcal bacteremia in adults. Clinical aspects and prognostic factors. Rev Med Rosario. 2008;74: 108–121.

18. Mayoral C, Baroni MR, Giani R, Virgolini S, Zurbriggen L, Regueira M. [Serotype distribution of Streptococcus pneumoniae isolated from invasive infections at the Hospital de Niños of Santa Fe]. Rev Argent Microbiol. 2008;40: 13–16.

19. Paganini H, Guiñazú JR, Hernández C, Lopardo H, Gonzalez F, Berberian G. Comparative analysis of outcome and clinical features in children with pleural empyema caused by penicillin-nonsusceptible and penicillin-susceptible Streptococcus pneumoniae. Int J Infect Dis. 2001;5: 86–88.

20. Palma I, Mosquera R, Demier C, Vay CA, Famiglietti A, Luna CM. Impact of bacteremia in a cohort of patients with pneumococcal pneumonia. J Bras Pneumol. 2012;38: 422–430.

21. Paniagua MM, Frisone HM, Romero JP, Merino DE. Meningitis in a pedriatic population in the province of Corrientes (Argentine). Prensa Med Argent. 2007;94: 367–373.

22. Pérez G, Mastroianni A, Parra A, Casimir L, Reijtman V, Lopardo H, et al. Infecciones invasivas con bacteriemia por Streptococcus pneumoniae en niños: ¿qué pasó en los últimos 5 años?^iesInvasive infections with bacteremia due to Streptococcus pneumoniae in children: What has happened over the past 5 years?^ien. Med Infant. 2014;21: 318–323.

23. Reijtman V, Fossati S, Hernandez C, Sommerfleck P, Bernaldez P, Litterio M, et al. Serotype distribution of streptococcus pneumoniae isolated from argentinian paediatric patients. Clin Microbiol Infect. 2011;17: S445.

24. Ruvinsky RO, Regueira M, Fossati MS, Gagetti P, Pace J, Rodriguez M, et al. Surveillance of invasive in Streptococcus pneumoniae in Argentina 1994-2007: Changes in serotype distribution, serotype coverage of pneumococcal conjugate vaccines and antibiotic resistance. Journal of Pediatric Infectious Diseases. 2010;5: 263–269.

25. Tregnaghi M, Ceballos A, Ruttimann R, Ussher J, Tregnaghi P, Peeters P, et al. Active epidemiologic surveillance of pneumonia and invasive pneumococcal disease in ambulatory and hospitalized infants in Cordoba, Argentina. Pediatr Infect Dis J. 2006;25: 370–372.

26. Zintgraff J, Fossati S, Pereira CS, Veliz O, Regueira M, Moscoloni MA, et al. Distribution of PCV13 and PPSV23 Streptococcus pneumoniae serotypes in Argentinean adults with invasive disease, 2013-2017. Rev Argent Microbiol. 2020;52: 189–194.

27. Alvares JR, Mantese OC, Paula A de, Wolkers PCB, Almeida VVP, Almeida SCG, et al. Prevalence of pneumococcal serotypes and resistance to antimicrobial agents in patients with meningitis: ten-year analysis. Braz J Infect Dis. 2011;15: 22–27.

28. Azevedo J, Dos Anjos ES, Cordeiro SM, Dos Santos MS, Escobar EC, Lobo PR, et al. Genetic profiles and antimicrobial resistance of Streptococcus pneumoniae non-PCV10 serotype isolates recovered from meningitis cases in Salvador,Brazil. J Med Microbiol. 2016;65: 1164–1170.

29. Barroso DE, Godoy D, Castiñeiras TM, Tulenko MM, Rebelo MC, Harrison LH. β-Lactam resistance, serotype distribution, and genotypes of meningitis-causing Streptococcus pneumoniae, Rio de Janeiro, Brazil. Pediatr Infect Dis J. 2012;31: 30–36.

30. Berezin EN, Falleiros-Carvalho LH, Lopes CR, Sanajotta AT, Brandileone MC, Menegatti S, et al. [Pneumococcal meningitis in children: clinical findings, most frequent serotypes and outcome]. J Pediatr. 2002;78: 19–23.

31. Berezin EN, Cardenuto MD, Ferreira LL, Otsuka M, Guerra ML, Brandileone MCC. Distribution of Streptococcus pneumoniae serotypes in nasopharyngeal carriage and in invasive pneumococcal disease in Sao Paulo, Brazil. Pediatr Infect Dis J. 2007;26: 643–645.

32. Berezin EN, Jarovsky D, Cardoso MRA, Mantese OC. Invasive pneumococcal disease among hospitalized children in Brazil before and after the introduction of a pneumococcal conjugate vaccine. Vaccine. 2020;38: 1740–1745.

33. Blanco BP, Branas PCAA, Yoshioka CRM, Ferronato AE. Pediatric bacterial meningitis and meningococcal disease profile in a Brazilian General Hospital. Braz J Infect Dis. 2020;24: 337–342.

34. Brandileone MCC, Sgambatti De Andrade ALS, Di Fabio JL, Guerra MLLS, Austrian R. Appropriateness of a pneumococcal conjugate vaccine in Brazil: Potential impact of age and clinical diagnosis, with emphasis on meningitis. J Infect Dis. 2003;187: 1206–1212.

35. Brandileone MCC, Almeida SCG, Bokermann S, Minamisava R, Berezin EN, Harrison LH, et al. Dynamics of antimicrobial resistance of Streptococcus pneumoniae following PCV10 introduction in Brazil: Nationwide surveillance from 2007 to 2019. Vaccine. 2021;39: 3207–3215.

36. Caierao J, Hawkins P, Sant’anna FH, Da Cunha GR, D’Azevedo PA, McGee L, et al. Serotypes and genotypes of invasive Streptococcus pneumoniaebefore and after PCV10 implementation in southern Brazil. PLoS One. 2014;9: A2433.

37. Camargos P, Nascimento-Carvalho CM, Teixeira R, França E. Lower respiratory infections mortality among Brazilians under-five before and after national pneumococcal conjugate vaccine implementation. Vaccine. 2020;38: 2559–2565.

38. de Pós- em Ciências da TA ao P, concentração: D. Características fenotípicas e moleculares de cepas de Streptococcus pneumoniae do sorotipo 19A isoladas de doenças invasivas e de portadores antes e após a introdução da vacina conjugada 10-valente no Brasil. [cited 11 Jul 2023]. Available: https://docs.bvsalud.org/biblioref/2022/09/1395390/ana-paula-cassiolato-tese-apc-defesa-final-ana-paula-cassiolato-1.pdf

39. Cazentini Medeiros MI, Grassi Almeida SC, Silva Guerra MLL, da Silva P, Machado Carneiro AM, de Andrade D, et al. Distribution of Streptococcus pneumoniae serotypes in the northeast macro-region of São Paulo state/Brazil after the introduction of conjugate vaccine. BMC Infect Dis. 2017;17: 1–9.

40. Christophe BL, Mott M, Da Cunha G, Caierao J, D’Azevedo P, Dias C. Characterisation of Streptococcus pneumoniae isolates from invasive disease in adults following the introduction of PCV10 in Brazil. J Med Microbiol. 2018;67: 687–694.

41. da Silva WA, Pinheiro AM, Coutinho LG, Marinho LAC, Lima LFA. Epidemiological profile of acute bacterial meningitis in the State of Rio Grande do Norte, Brazil. Rev Soc Bras Med Trop. 2010;43: 455–457.

42. Dos Santos SR, Passadore LF, Takagi EH, Fujii CM, Yoshioka CRM, Gilio AE, et al. Serotype distribution of Streptococcus pneumoniae isolated from patients with invasive pneumococcal disease in Brazil before and after ten-pneumococcal conjugate vaccine implementation. Vaccine. 2013;31: 6150–6154.

43. Magalhaes APG de O, Pinto A da S. Antimicrobial resistance and serotyping of Streptococcus pneumoniae isolated from pediatric patients in Belo Horizonte, MG, Brazil. Braz J Microbiol. 2003;34: 210–212.

44. Gouveia EL, Reis JN, Flannery B, Cordeiro SM, Lima JBT, Pinheiro RM, et al. Clinical outcome of pneumococcal meningitis during the emergence of pencillin-resistant Streptococcus pneumoniae: An observational study. BMC Infect Dis. 2011; 323.

45. Hirose TE, Maluf EMCP, Rodrigues CO. Pneumococcal meningitis: epidemiological profile pre- and post-introduction of the pneumococcal 10-valent conjugate vaccine. J Pediatr. 2015;91: 130–135.

46. Jarovsky D, Berezin EN, De Almeida RJS. Invasive pneumococcal disease in a population with underlying comorbidities. Open Forum Infectious Diseases. 2017;4: S464.

47. Laval CB, de Andrade ALSS, Pimenta FC, de Andrade JG, de Oliveira RM, Silva SA, et al. Serotypes of carriage and invasive isolates of Streptococcus pneumoniae in Brazilian children in the era of pneumococcal vaccines. Clin Microbiol Infect. 2006;12: 50–55.

48. Leite CR, Azevedo J, Galvao VS, Moreno-Carvalho O, Reis JN, Nascimento-Carvalho C. Clinical and bacteriological characteristics of invasive pneumococcal disease after pneumococcal 10-valent conjugate vaccine implementation in Salvador, Brazil. Braz J Infect Dis. 2016;20: 56–60.

49. Levin AS, Sessegolo JF, Teixeira LM, Barone AA. Factors associated with penicillin-nonsusceptible pneumococcal infections in Brazil. Braz J Med Biol Res. 2003;36: 807–813.

50. Mantese OC, Hirano J, Santos IC, Silva VM, De Castro E. Etiological profile of bacterial meningitis in children. J Pediatr. 2002;78: 467–474.

51. Mantese OC, De Paula A, Almeida VVP, De Aguiar PADF, Wolkers PCB, Alvares JR, et al. Prevalence of serotypes and antimicrobial resistance of invasive strains of pneumococcus in children: Analysis of 9 years. J Pediatr. 2009;85: 495–502.

52. Lages PM, Carlesse F, Boettger BC, Pignatari ACC, Petrilli AS, de Moraes-Pinto MI. Invasive pneumococcal disease in children with cancer: Incidence density, risk factors and isolated serotypes. Braz J Infect Dis. 2020;24: 489–496.

53. Menezes APDO, Campos LC, dos Santos MS, Azevedo J, dos Santos RCN, Carvalho MDGS, et al. Serotype distribution and antimicrobial resistance of Streptococcus pneumoniae prior to introduction of the 10-valent pneumococcal conjugate vaccine in Brazil, 2000-2007. Vaccine. 2011;29: 1139–1144.

54. Mott M, Caierao J, Rosa da Cunha G, Rodrigues Perez LR, Matusiak R, Pilger de Oliveira KR, et al. Susceptibility profiles and correlation with pneumococcal serotypes soon after implementation of the 10-valent pneumococcal conjugate vaccine in Brazil. Int J Infect Dis. 2014;20: 47–51.

55. Nascimento-Carvalho CM, Freitas-Souza LS, Moreno-Carvalho OA, Alves NN, Caldas RM, Barberino MG, et al. Invasive pneumococcal strains isolated from children and adolescents in Salvador. J Pediatr. 2003;79: 209–214.

56. Reis JN, Cordeiro SM, Coppola SJ, Salgado K, Carvalho MGS, Teixeira LM, et al. Population-based survey of antimicrobial susceptibility and serotype distribution of Streptococcus pneumoniae from meningitis patients in Salvador, Brazil. J Clin Microbiol. 2002;40: 275–277.

57. Novaes HMD, Sartori AMC, de Soarez PC. Hospitalization rates for pneumococcal disease in Brazil, 2004 - 2006. Rev Saude Publica. 2011;45: 539–547.

58. Oliveira DS, Chiaravalloti Neto F, Mota TS, Araujo DB, Sartori AMC. Spatial analysis of pneumococcal meningitis in São Paulo in the pre- and post-immunization era. Rev Saude Publica. 2019;53: 59.

59. Pinto TCA, Neves FPG, Souza ARV, Oliveira LMA, Costa NS, Castro LFS, et al. Evolution of penicillin non-susceptibility among streptococcus pneumoniae isolates recovered from asymptomatic carriage and invasive disease over 25 years in Brazil, 1990-2014. Front Microbiol. 2019;10: 486.

60. Dullius CR, Zani L, Chatkin JM. Theoretical pneumococcal vaccine coverage: analysis of serotypes isolated from inpatients at a tertiary care hospital^ienCobertura vacinal pneumocócica teórica: análise de sorotipos isolados de pacientes internados em hospital terciário^ipt. J Bras Pneumol. 2018;44: 361–366.

61. Rossoni AM de O, Costa LMD, Berto DB, Farah SS, Gelain M, Brandileone MC de C, et al. Acute bacterial meningitis caused by Streptococcus pneumoniae resistant to the antimicrobian agents and their serotypes. Arq Neuropsiquiatr. 2008;66: 509–515.

62. Temporal trends and clonal diversity of penicillin non-susceptible pneumococci from meningitis cases from 1996 to 2012, in Salvador, Brazil. 2015. pp. 1–10. doi:10.1186/s12879-015-1049-y

63. Veras MASM, Enanoria WTA, Castilho EA, Reingold AL. Effectiveness of the polysaccharide pneumococcal vaccine among HIV-infected persons in Brazil: A case control study. BMC Infect Dis. 2007;7: 119.

64. Vieira AC, Gomes MC, Rolo Filho M, Eudes Filho J, Bello EJM, De Figueiredo RB. Streptococcus pneumoniae: A study of strains isolated from cerebrospinal fluid. J Pediatr. 2007;83: 71–78.

65. Yoshioka CRM, Martinez MB, Brandileone MCC, Ragazzi SB, Guerra MLLS, Santos SR, et al. Analysis of invasive pneumonia-causing strains of Streptococcus pneumoniae: Serotypes and antimicrobial susceptibility. J Pediatr. 2011;87: 70–75.

66. Cristian Aguilera R, Gerardo Gonzalez R, Helia Bello T, Sergio Mella M, Rodrigo Blamey D, Henriette Chabouty G, et al. Antimicrobial susceptibility, capsular serotypes and clonal relationship of invasive Streptococcus pneumoniae isolates in adult population of the Bio-Bio Region, Chile. 2005-2006. Rev Chilena Infectol. 2010;27: 392–397.

67. Alvarado S, Cavada G, Villena R, Wilhelm J, Budnik I, Lara C, et al. Efecto de la vacuna antineumocócica conjugada 10-valente en el área sur de Santiago de Chile, 2009-2015. Rev Panam Salud Publica. 2018;42: 1–7.

68. Contreras L, Fica A, Figueroa O, Enriquez N, Urrutia P, Herrera P. Resistance to Streptococcus pneumoniae to penicillin and its association with clinical and epidemiological factors. Rev Med Chil. 2002;130: 26–34.

69. Fica A, Bunster N, Aliaga F, Olivares F, Porte L, Braun S, et al. Bacteremic pneumococcal pneumonia: serotype distribution, antimicrobial susceptibility, severity scores, risk factors, and mortality in a single center in Chile^ien. Braz J Infect Dis. 2014;18: 115–123.

70. Inostroza J, Illesca V, Reydet P, Vinet AM, Ossa G, Munoz S, et al. Ten-year surveillance of pneumococcal infections in Temuco, Chile: Implications for vaccination strategies. Clin Vaccine Immunol. 2007;14: 660–664.

71. Katia Abarca V, Rodrigo Vergara F, Enzo Tassara P, Isabel Ibanez W, Cristian Garcia B, Marcela Potin S. Invasive pneumococcal disease and consolidated pneumonia in infants: One year of surveillance in three Chilean health care centers. Rev Chilena Infectol. 2008;25: 97–103.

72. Lagos R, Muñoz A, San Martin O, Maldonado A, Hormazabal JC, Blackwelder WC, et al. Age- and serotype-specific pediatric invasive pneumococcal disease: insights from systematic surveillance in Santiago, Chile, 1994--2007. J Infect Dis. 2008;198: 1809–1817.

73. Maldonado BA, Seoane MM, San Martin BO, Hormazabal OJC, Lagos Zuccone R. Retrospective assessment of laboratory surveillance for invasive Streptococcus pneumoniae isolates from adults of the Metropolitan Region (Chile): 2000-2006. Rev Chilena Infectol. 2007;24: 446–452.

74. Rioseco ML, Riquelme R. [Bacteremic pneumococcal pneumonia in 45 immunocompromised hospitalized adults]. Rev Med Chil. 2004;132: 588–594.

75. Rioseco Z ML, Riquelme O R, Riquelme O M, Inzunza P C, Riquelme D J, Sanhueza R A. Neumonía neumocócica bacteriémica en adultos en hospital regional de Chile^iesBacteremic pneumococcal pneumonia in adults admitted to a general hospital: experience in 60 cases^ien. Rev méd Chile. 2018;146: 839–845.

76. Saldías F, Díaz O. [Severity scores for predicting clinically relevant outcomes for immunocompetent adult patients hospitalized with community-acquired pneumococcal pneumonia]. Rev Chilena Infectol. 2011;28: 303–309.

77. Valenzuela MT, Seoane M, Canals A, Pidal P, Hormazábal JC, Araya P, et al. Vigilancia de laboratorio de Streptococcus pneumoniae procedente de enfermedad invasora, Chile 2007-2012^iesLaboratory surveillance of Streptococcus pneumoniae from invasive disease, Chile 2007-2012^ien. Rev Chilena Infectol. 2014;31: 651–658.

78. Africano HF, Serrano-Mayorga CC, Ramirez-Valbuena PC, Bustos IG, Bastidas A, Vargas HA, et al. Major adverse cardiovascular events during invasive pneumococcal disease are serotype dependent. Clin Infect Dis. 2020;72: e711–e719.

79. Agudelo CI, Sanabria OM, Ovalle MV, Castaneda E. Laboratory surveillance of Streptococcus pneumoniae serotypes from infected children aged less than 5 years: 1994-2000. Vigilancia por el laboratorio de Streptococcus pneumoniae, aislado de procesos invasores en ninos menores de 5 anos: actualizacion de los datos 1994-2000. Biomédica 2001;21: 193–199.

80. Ines Agudelo C, Lucia Diaz P, Marina Sanabria O, Victoria Ovalle M, Castaneda E, Rosa Gallego C, et al. Laboratory surveillance of Streptococcus pneumoniae isolated by invasive processes in the population older than 5 years, 1998-2001. Vigilancia por el laboratorio de Streptococcus pneumoniae aislado de procesos invasores en poblacion mayor de 5 anos, 1998-2001.CABI databases 2002;7: 177–183. https://www.cabidigitallibrary.org/doi/full/10.5555/20023161394 Accessed May 5th 2023

81. Agudelo CI, Moreno J, Sanabria OM, Ovalle MV, Di Fabio JL, Castaneda E. Streptococcus pneumoniae: serotype evolution and patterns of antimicrobial susceptibility in invasive isolates from 11 years surveillance (1994 -2004) in Colombia. Biomedica. 2006;26: 234–249.

82. Caceres DC, Ortega-Barria E, Nieto J, DeAntonio R. Pneumococcal meningitis trends after pneumococcal conjugate vaccine introduction in Colombia: An interrupted time-series analysis. Hum Vaccin Immunother. 2018;14: 1230–1233.

83. Calderón C, Dennis R. Costos económicos de neumonía adquirida en comunidad, meningitis y bacteriemia por Streptococcus pneumoniae en una población adulta que requirió hospitalización en Bogotá, Colombia^iesEconomic cost of Streptococcus pneumoniae community-acquired pneumonia,. Biomédica (Bogotá). 2014;34: 92–101.

84. Camacho Moreno G, Imbachi LF, Leal AL, Moreno VM, Patino JA, Gutierrez IF, et al. Emergence of Streptococcus pneumoniae serotype 19A (Spn19A) in the pediatric population in Bogota, Colombia as the main cause of invasive pneumococcal disease after the introduction of PCV10. Hum Vaccin Immunother. 2020;16: 2300–2306.

85. Farfan-Albarracin JD, Camacho-Moreno G, Leal AL, Patino J, Coronell W, Gutierrez IF, et al. Changes in the incidence of acute bacterial meningitis caused by Streptococcus pneumoniae and the implications of serotype replacement in children in Colombia after mass vaccination with PCV10. Frontiers in Pediatrics. 2022;10: 1006887.

86. GutiÃ©rrez-Tobar IF, Londoño-Ruiz JP, Mariño-Drews C, Beltrán-Higuera S, Camacho-Moreno G, Leal-Castro AL, et al. Epidemiological characteristics and serotype distribution of culture-confirmed pediatric pneumococcal pneumonia before and after PCV 10 introduction, a multicenter study in Bogota, Colombia, 2008â€“2019. Vaccine. 2022;40: 2875–2883.

87. Castro ALL, Camacho-Moreno G, Montañez-Ayala A, Varón-Vega F, Alvarez-Rodríguez JC, Valderrama-Beltrán S, et al. Invasive Pneumococcal Disease Characterization in Adults and Subgroups aged < 60 years and ≥ 60 years in Bogota, Colombia. IJID Reg. 2022;3: 293–299.

88. Moreno J, Phandanouvong V, Castaneda E. Molecular surveillance of invasive penicillin-resistant Streptococcus pneumoniae Colombian isolates recovered from children less than 5 years of age. Biomedica. 2004;24: 296–301.

89. Narváez PO, Gomez-Duque S, Alarcon JE, Ramirez-Valbuena PC, Serrano-Mayorga CC, Lozada-Arcinegas J, et al. Invasive pneumococcal disease burden in hospitalized adults in Bogota, Colombia. BMC Infect Dis. 2021;21: 1–12.

90. Parra EL, Ramos V, Sanabria O, Moreno J. Serotype and genotype distribution among invasive Streptococcus pneumoniae isolates in Colombia, 2005-2010. PLoS One. 2014;9: e84993.

91. Parra EL, Duarte C, Rodriguez K, Sanabria O, Moreno J. Frequency and molecular characterization of invasive isolates of Streptococcus pneumoniae serotypes 6C and 6D in Colombia. Enferm Infecc Microbiol Clin. 2017;35: 283–286.

92. Rojas JP, Leal AL, Patiño J, Montañez A, Camacho G, Beltrán S, et al. Caracterización de pacientes fallecidos por enfermedad neumocóccica invasiva en la poblacion infantil de Bogotá, Colombia^iesCharacterization of patients who died of invasive pneumococcal disease in the child population of Bogota, Colombia^ien. Rev Chil Pediatr. 2016;87: 48–52.

93. Severiche-Bueno DF, Bastidas A, Caceres EL, Silva E, Lozada J, Gomez S, et al. Burden of invasive pneumococcal disease (IPD) over a 10-year period in Bogotá, Colombia. Int J Infect Dis. 2021;105: 32–39.

94. Vela MC, Fonseca N, Di Fabio JL, Castaneda E. Presence of international multiresistant clones of Streptococcus pneumoniae in Colombia. Microb Drug Resist. 2001;7: 153–164.

95. Barboza C, Brenes H, Avila-Aguero ML, Avila L, Camacho K. Epidemiology of bacterial meningitis in pediatric population after the introduction of pneumococcal conjugated vaccine in costa rica. Open Forum Infectious Diseases. 2018;5: S138.

96. Ulloa-Gutierrez R, Avila-Aguero ML, Herrera ML, Herrera JF, Arguedas A. Invasive pneumococcal disease in Costa Rican children: A seven year survey. Pediatr Infect Dis J. 2003;22: 1069–1074.

97. Vargas-Gutierrez M, Guila-Esquivel E, Vargas-Acuna MT, Ulloa-Gutierrez R, Soto-Martinez ME. Hospitalizations due to complicated pneumonia with parapneumonic pleural effusion or empyema in Costa Rican children: A 9-year study. Am J Respir Crit Care Med. 2015;191. Available: http://ovidsp.ovid.com/ovidweb.cgi?T=JS&PAGE=reference&D=emed16&NEWS=N&AN=72050463

98. Batista Caluff L, González Fernández N, Donatién Rojas NC, Cobas Limonta N, Jústiz Hernández S, Herrera López J. Enfermedad neumocócica en menores de 5 años en el Hospital Infantil Norte de Santiago de Cuba “Dr. Juan de la Cruz Martínez Maceira”^iesPneumococcal disease seen in children younger than 5 years in “Juan de la Cruz Martinez Maceira” Northern Pediatric Hos. Rev Cubana Pediatr. 2017;89: 144–155.

99. Dickinson Meneses F, Rodríguez Ortega M. Epidemiología de la meningitis neumocócica en niños cubanos menores de 6 años^iesEpidemiology of pneumococcal meningitis in Cuban children younger than 6 years^ien. Rev Cubana Pediatr. 2017;89: 52–64.

100. Fonseca Hernández M, Martínez Utrera A, Montes de Oca Rivero M, Cardoso Hernández E, Reyes Sebasco A, Llull Tombo CT, et al. Enfermedad neumocócica invasiva en niños menores de 6 años hospitalizados^iesInvasive pneumococcal disease in hospitalized children aged less than 6 years^ien. Rev Cubana Pediatr. 2017;89: 133–143.

101. Alvarez OM, Jimenez DM, Hernandez MF, Utrera AM. Invasive pneumococcal disease in children younger than five years in Cienfuegos (2009-2015). Medisur-Rev Cienc Med Cienfuegos. 2019;17: 494–504.

102. Perez AE, Dickinson FO, Rodriguez M. Community acquired bacterial meningitis in Cuba: A follow up of a decade. BMC Infect Dis. 2009;10: 130.

103. Rodríguez Cutting JM, Vega Mendoza D, Pacheco Torres L, Piedra Bello M, García Sánchez JB, Del Valle Rodríguez R. Características clínicas e imaginológicas de niños con neumonía complicada causada por Streptococcus pneumoniae^iesClinical and imaging characteristics of children with complicated pneumonia caused by Streptococcus pneumoniae^ien. Rev Cubana Pediatr. 2017;89: 65–76.

104. Toraño-Peraza G, Puas-Solis L, Abreu-Capote M, Rodriguez-Ortega M, Dickinson-Meneses F, Varcarcel-Sanchez M. Serotipos y resistencia antimicrobiana de aislamientos meníngeos de Streptococcus pneumoniae. Cuba, 2007-2012. Vaccimonitor. 2014;23: 117–123.

105. Toraño Peraza G, Suárez Aspaza D, Abreu Capote M, Barreto Núnez B, Toledo Romaní E, Linares Pérez N. Serotipos de Streptococcus pneumoniae responsables de enfermedad invasiva en niños cubanos^ies. Rev Cubana Pediatr. 2017;89: 172–180.

106. Ahmed SS, Lessa FC, Coradin H, Sánchez J, Carvalho M da G, Soda E, et al. High Prevalence of Vaccine-Type Infections Among Children with Pneumococcal Pneumonia and Effusion After 13-Valent Pneumococcal Conjugate Vaccine Introduction in the Dominican Republic. J Infect Dis. 2021;224: S228–S236.

107. Jonnalagadda S, Rodriguez O, Estrella B, Sabin LL, Sempertegui F, Hamer DH. Etiology of severe pneumonia in Ecuadorian children. PLoS One. 2017;12: e0171687.

108. Juliao P, Guzman-Holst A, Gupta V, Velez C, Rosales T, Torres C. Incidence and Mortality Trends of Acute Gastroenteritis and Pneumococcal Disease in Children Following Universal Rotavirus and Pneumococcal Conjugate Vaccination in Ecuador. Infectious Diseases and Therapy. 2021;10: 2593–2610.

109. Elenga N, Sicard S, Cuadro-Alvarez E, Long L, Njuieyon F, Martin E, et al. Pediatric bacterial meningitis in French Guiana. Med Mal Infect. 2015;45: 441–445.

110. Gaensbauer JT, Asturias EJ, Soto M, Holt E, Olson D, Halsey NA, et al. Pediatric Invasive Pneumococcal Disease in Guatemala City: Importance of Serotype 2. Pediatr Infect Dis J. 2016;35: e139–e143.

111. Melgar M, Maldonado H, Contreras I, Contreras CL, López MR, MacCracken J, et al. Enfermedad Neumocóccica Invasiva y Neumonía Adquirida en la Comunidad Radiológicamente Confirmada en dos hospitales de referencia de ciudad de Guatemala, resultados de 3 años de vigilancia. 18 Congreso Latinoamericano de Infectología Pediátrica. 2019.

112. Trotman H, Olugbuyi O, Barton M, McGregor D, Thomas S. Pneumococcal meningitis in Jamaican children. West Indian Med J. 2009;58: 585–588.

113. Willis R, Heslop O, Bodonaik N, Thame M, Kumar U, Aung M, et al. Pneumococcal disease burden, clinical presentations and vaccine coverage in the Jamaican population. Hum Antibodies. 2018;26: 193–199.

114. Willis R, Heslop O, Bodonaik N, Thame M, Smikle M. Morbidity, mortality and antimicrobial resistance of pneumococcal infections in the Jamaican paediatric and adult populations. Hum Antibodies. 2019;27: 155–160.

115. Cardoso MRA, Nascimento-Carvalho CM, Ferrero F, Berezin EN, Ruvinsky R, Camargos PAM, et al. Penicillin-resistant pneumococcus and risk of treatment failure in pneumonia. Arch Dis Child. 2008;93: 221–225.

116. Andrade AL, Arguedas A, Benavides J, Minamisava R, De Brandileone MCC, Soley C, et al. Bacteriology of community-acquired invasive disease found in a multicountry prospective, population-based, epidemiological surveillance for pneumococcus in children in Latin America. Pediatr Infect Dis J. 2012;31: 1312–1314.

117. Hortal M, Ruvinsky R, Rossi A, Agudelo CI, Castañeda E, Brandileone C, et al. [Impact of Streptococcus pneumoniae on pneumonia in Latin American children. SIREVA-Vigía Group]. Rev Panam Salud Publica. 2000;8: 185–195.

118. Moreno J, Duarte C, Cassiolato AP, Chacon GC, Alarcon P, Sanchez J, et al. Molecular characterization of Latin American invasive Streptococcus pneumoniae serotype 19A isolates. Vaccine. 2020;38: 3524–3530.

119. Peltola H, Roine I, Kallio M, Pelkonen T. Outcome of childhood bacterial meningitis on three continents. Sci Rep. 2021;11: 21593.

120. Zemlickova H, Crisostomo MI, Brandileone MC, Camou T, Castaneda E, Corso A, et al. Serotypes and clonal types of penicillin-susceptible Streptococcus pneumoniae causing invasive disease in children in five Latin American countries. Microb Drug Resist. 2005;11: 195–204.

121. Arredondo-Garcia JL, Calderon E, Echaniz-Aviles G, Soto-Nogueron A, Arzate P, Amabile-Cuevas CF. Serotypes and antibiotic susceptibility of Streptococcus pneumoniae isolates causative of invasive diseases in Mexican children. J Infect Dev Ctries. 2011;5: 119–122.

122. Chacon-Cruz E, Alvelais-Palacios JA, Lopatynsky-Reyes EZ, Volker-Soberanes ML, Rivas-Landeros RM. Continuous pneumococcal invasive disease reduction, high impact on pneumococcal empyemas and meningitis, and serotype 19A Disappearance Following PCV-13 Vaccination in Children: Eleven and a Half Years of Active Surveillance in a Mexican Hospital on the U. Open Forum Infectious Diseases. 2017;4: S680.

123. Chacon-Cruz E, Rivas-Landeros RM, Volker-Soberanes ML, Lopatynsky-Reyes EZ, Becka C, Alvelais-Palacios JA. 12 years active surveillance for pediatric pleural empyema in a Mexican hospital: effectiveness of pneumococcal 13-valent conjugate vaccine, and early emergence of methicillin-resistant Staphylococcus aureus. Therapeutic Advances in Infectious Disease. 2019;6. doi:10.1177/2049936119839312

124. Echaniz-Aviles G, Soto-Nogueron A, Miranda-Novales G, Carnalla-Barajas MN, Velazquez-Meza ME, Solorzano-Santos F, et al. Streptococcus pneumoniae serotypes identified in Mexican children with invasive disease before and after the introduction of PCV7 (1993-2012). Arch Med Res. 2015;46: 149–153.

125. Echaniz-Aviles G, Garza-Gonzalez E, Roman-Mancha AL, Morfin-Otero R, Rodriguez-Noriega E, Ayala-Gaytan JJ, et al. Clinical and microbiological characteristics of community-acquired pneumonia associated with Streptococcus pneumoniae in adult patients in Mexico. Rev Argent Microbiol. 2019;51: 234–240.

126. Franco-Paredes C, Lammoglia L, Hernandez I, Santos-Preciado JI. Epidemiology and outcomes of bacterial meningitis in Mexican children: 10-year experience (1993-2003). Int J Infect Dis. 2008;12: 380–386.

127. Gomez-Barreto D, Espinosa-Monteros LE, Lopez-Enriquez C, Jimenez-Rojas V, Rodriguez-Suarez R. Invasive pneumococcal disease in a third level pediatric hospital in Mexico City: Epidemiology and mortality risk factors. Salud Publica Mex. 2010;52: 391–397.

128. Zarco-Marquez S, Volkow-Fernandez P, Velazquez-Acosta C, Echaniz-Aviles G, Carnalla-Barajas MN, Soto-Nogueron A, et al. Invasive and complicated pneumococcal infection in patients with cancer. Revista de Investigacion Clinica - Clinical and Translational Investigation. 2016;68: 221–228.

129. De León T, Daza C, Cukier G, Chong E, Saldaña R, Samudio Castillo R. Impacto del serotipo 5 en la enfermedad invasora por Streptococcus pneumoniae en la población pediátrica ingresada en el Hospital Materno Infantil José Domingo de Obaldía^ies. Pediátr Panamá. 2011;40: 7–15.

130. Aranda C, Lovera D, Arbo A. Cambios en el Patrón Epidemiológico y resistencia Bacteriana de la Meningitis Bacteriana Aguda en Niños en un hospital de referencia^ies. Rev Inst Med Trop Sao Paulo. 2014;9.

131. León ME, Kawabata A, Nagai M, Rojas L, Zárate N, Irala J, et al. Frecuencia de Streptococcus pneumoniae aislados de enfermedad invasiva en Paraguay, serotipos y perfil de sensibilidad (2010-2018)^iesFrequency of Streptococcus pneumoniae isolated from invasive disease in Paraguay, serotypes and sensitivity profile (2010. Mem Inst Invest Cienc Salud (Impr). 2020;18.

132. Lovera D, Aranda C, Duarte M, Apodaca S, Acuña J, Arbo A. Predicción de la mortalidad de la meningitis neumocóccica en niños^iesPredicting mortality from pneumococcal meningitis in children^ien. Pediatr (Asunción). 2011;38: 111–117.

133. Sanabria G, Araya S, Chamorro G, Lovera D, Arbo A. Correlación de serotipos, sensibilidad y resistencia antimicrobiana en niños con infecciones invasivas por Streptococcus pneumoniae en un centro de referencia de Asunción-Paraguay. Revisión de 6 años^ies. Rev Inst Med Trop Sao Paulo. 2009;4.

134. Castro JD, Siccha SM, Egoavil M, Chaparro E, Hernandez R, Silva W, et al. Resistencia antibiótica y distribución de serotipos en cepas neumocócicas invasivas en adultos hospitalizados en Lima, Perú^iesAntibiotic resistance and distribution of serotypes of invasive pneumococcal strains isolated from hospitalized adults in Lima,. Rev Peru Med Exp Salud Publica. 2017;34: 633–641.

135. Castillo-Tokumori F, Mercado E, Marcelo M, Del Aguila O, Reyes I, Campos F, et al. Demographic and clinical characteristics of children with invasive pneumococcal disease in Lima, Peru. Am J Trop Med Hyg. 2018;99: 630.

136. Hawkins P, Mercado E, Chochua S, Castillo ME, Reyes I, Chaparro E, et al. Key features of invasive pneumococcal isolates recovered in Lima, Peru determined through whole genome sequencing. Int J Med Microbiol. 2017;307: 415–421.

137. Luna-Muschi A, Castillo-Tokumori F, Deza MP, Mercado EH, Egoavil M, Sedano K, et al. Invasive pneumococcal disease in hospitalised children from Lima, Peru before and after introduction of the 7-valent conjugated vaccine. Epidemiol Infect. 2019;147: e91.

138. Instituto Nacional de Salud . Grupo Multifuncional de, Neumonias. Vigilancia epidemiológica centinela de Haemophilus influenzae y Streptococcus pneumoniae en menores de 5 años en el Perú^ies. Rev Peru Med Exp Salud Publica. 2003;20: 150–155.

139. Rivera-Matos IR, Rios-Olivares E. A multicenter hospital surveillance of invasive Streptococcus pneumoniae, Puerto Rico, 2001. P R Health Sci J. 2005;24: 185–189.

140. Nurse-Lucas M, McGee L, Hawkins PA, Swanston WH, Akpaka PE. Serotypes and genotypes of Streptococcus pneumoniae isolates from Trinidad and Tobago. Int J Infect Dis. 2016;46: 100–106.

141. Assandri E, Amorín B, Gesuele JP, Algorta G, Pírez MC. Enfermedad neumoccócica invasora en recién nacidos, antes y después de la vacunación universal con vacuna conjugada 7 y 13 valente en Uruguay^iesPneumococcal invasive disease in newborns before and after 7-valent and 13-valent universal pneumococcal vacci. Rev Chilena Infectol. 2015;32: 167–174.

142. Camou T, Palacio R, Di Fabio JL, Hortal M. Invasive pneumococcal diseases in Uruguayan children: Comparison between serotype distribution and conjugate vaccine formulations. Vaccine. 2003;21: 2093–2096.

143. Cardinal-Fernandez P, Garcia Gabarrot G, Echeverria P, Zum G, Hurtado J, Rieppi G. Clinical and microbiological aspects of acute community-acquired pneumonia due to Streptococcus pneumoniae. Aspectos clinicos y microbiologicos de la neumonia aguda comunitaria a Streptococcus pneumoniae. 2013;213: 88–96.

144. Ferrari Castilla AM, Pirez GMC, Martinez AA, Algorta RG, Chamorro VF, Guala BMJ, et al. Etiology of community acquired pneumonia in inpatients children. Uruguay 1998-2004. Rev Chilena Infectol. 2007;24: 40–47.

145. Gabarrot GG, Vega MLP, Giffoni GPR, Ndez SH, Cardinal P, Lix VF, et al. Effect of pneumococcal conjugate vaccination in uruguay, a middle-income Country. PLoS One. 2014;9: e112337.

146. Hortal M, Camou T, Palacio R, Dibarboure H, Garcia A. Ten-year review of invasive pneumococcal diseases in children and adults from Uruguay: Clinical spectrum, serotypes, and antimicrobial resistance. Int J Infect Dis. 2000;4: 91–95.

147. Hortal M, Estevan M, Iraola I, De Mucio B. A population-based assessment of the disease burden of consolidated pneumonia in hospitalized children under five years of age. Int J Infect Dis. 2007;11: 273–277.

148. Hortal M, Sehabiague G, Camou T, Iraola I, Estevan M, Pujadas M. Pneumococcal pneumonia in hospitalized Uruguayan children and potential prevention with different vaccine formulations. J Pediatr. 2008;152: 850–853.

149. Hortal M, Estevan M, Meny M, Iraola I, Laurani H. Impact of pneumococcal conjugate vaccines on the incidence of pneumonia in hospitalized children after five years of its introduction in Uruguay. PLoS One. 2014;9: e98567.

150. Machado K, López A, Pacheco H, Algorta G, Pírez C. Características del empiema paraneumónico luego del inicio de la vacunación antineumocócica: Centro Hospitalario Pereira Rossell, año 2010^iesFeatures of parapneumonic empyema after the beginning of pneumococcal vaccination: CHPR, 2010^ien. Arch Pediatr Urug. 2014;85: 212–2219.

151. Machado K, Badía F, Assandri E, Gutiérrez C, Motta I, Varela A, et al. Neumonía necrotizante en niños: 10 años de experiencia en un hospital pediátrico de referencia^iesNecrotizing pneumonia in children: 10 years of experience in a Pediatric Reference Hospital^ienPneumonia necrosante em crianças: 10 anos de experiência em um. Arch Pediatr Urug. 2020;91: 294–302.

152. Pirez MC, Martinez O, Ferrari AM, Nairac A, Rubio IAMO, Sarachaga MJ, et al. Standard case management of pneumonia in hospitalized children in Uruguay, 1997 to 1998. Pediatr Infect Dis J. 2001;20: 283–289.

153. Pirez MC, Algorta G, Chamorro F, Romero C, Varela A, Cedres A, et al. Changes in hospitalizations for pneumonia after universal vaccination with pneumococcal conjugate vaccines 7/13 valent and haemophilus influenzae type b conjugate vaccine in a pediatric referral hospital in Uruguay. Pediatr Infect Dis J. 2014;33: 753–759.

154. Pirez MC, Mota MI, Giachetto G, Sanchez Varela M, Galazka J, Gutierrez S, et al. Pneumococcal Meningitis before and after Universal Vaccination with Pneumococcal Conjugate Vaccines 7/13, Impact on Pediatric Hospitalization in Public and Nonpublic Institutions, in Uruguay. Pediatr Infect Dis J. 2017;36: 1000–1001.

155. Pirez Garcia MC, Giachetto Larraz G, Romero Rostagno C, Zabala Chain C, Algorta Rusinol G, Montano Lotito A, et al. Invasive pneumococcal pneumonia in children 0-24 months old: Does bacterial resistance affect outcome? An Pediatr. 2008;69: 205–209.
