# Supplementary material for: Invasive pneumococcal disease in Latin America and the Caribbean: Serotype distribution, disease burden, and impact of vaccination. A systematic review and meta-analysis
Source: PLoS One. 2024 Jun 27;19(6):e0304978. doi: 10.1371/journal.pone.0304978 (PMC11210815; doi:10.1371/journal.pone.0304978)
Supplement: S3 File — (DOCX) [file pone.0304978.s004.docx]

# **Supporting information - Figures**

**S4 Figure 1: Percentage and number of serotypes by vaccine type, non-vaccine, and non-typable isolates per year and country.**

**S4 Figure 2: Isolates per 100,000 population, for 2006-2018 in seven LAC countries by age groups.**

**S4 Figure 3: Vaccine serotypes before and after introducing PCV10 or PCV13 vaccines in seven countries.**

*** CAREC (Caribbean Epidemiology Center)**

**S2 Figure 1: Percentage and number of serotypes by vaccine type, non-vaccine, and non-typable isolates per year and country**

**S2 Figure 2: Isolates per 100,000 population, for 2006-2018 in seven LAC countries by age groups.**

**S2 Figure 3: Vaccine serotypes before and after introducing PCV10 or PCV13 vaccines in seven countries.**
