# Supplementary material for: Invasive pneumococcal disease in Latin America and the Caribbean: Serotype distribution, disease burden, and impact of vaccination. A systematic review and meta-analysis
Source: PLoS One. 2024 Jun 27;19(6):e0304978. doi: 10.1371/journal.pone.0304978 (PMC11210815; doi:10.1371/journal.pone.0304978)
Supplement: Attachment — Submitted filename: Response to reviewers R2.docx [file pone.0304978.s006.docx]

Response to Reviewers R2:

Please follow reviewer# 2 suggestion to move Table 1 to supplementary information.

Thank you. Done

Please read PlosOne policy on reference/citations. I am not sure you can cite a study/abstract/poster if it is not availabe in the public domain.

Thank you, we added more information and links to these 3 references:

Benitez JD, Martinez ME, Specht MH von, Gerlach E, Gonzalez CA, Grenon SL. Epidemiology and risk factors for invasive pneumococcal disease in pediatrics. Descriptive, postvaccinal study. Epidemiología y factores de riesgo de enfermedad invasiva neumocócica en pediatría Estudio descriptivo, postvacunal. Revista de ciencia y tecnologia 2017; 4–10. Available: <http://ri.conicet.gov.ar/handle/11336/177141 Accessed May 5th 2023>

Agudelo CI, Sanabria OM, Ovalle MV, Castaneda E. Laboratory surveillance of Streptococcus pneumoniae serotypes from infected children aged less than 5 years: 1994-2000. Biomédica 2001;21(2): 193–199.

Agudelo I, Diaz LP, Sanabria MO, Ovalle VM, Castaneda E, Rosa Gallego C, et al. Laboratory surveillance of Streptococcus pneumoniae isolated by invasive processes in the population older than 5 years, 1998-2001. Vigilancia por el laboratorio de Streptococcus pneumoniae aislado de procesos invasores en población mayor de 5 años, 1998-2001. 2002;7: 177–183. <https://www.cabidigitallibrary.org/doi/full/10.5555/20023161394>
Accessed May 5th 2023
